# Geography, Ethnicity or Subsistence-Specific Variations in Human Microbiome Composition and Diversity

**DOI:** 10.3389/fmicb.2017.01162

**Published:** 2017-06-23

**Authors:** Vinod K. Gupta, Sandip Paul, Chitra Dutta

**Affiliations:** ^1^Structural Biology and Bioinformatics Division, CSIR-Indian Institute of Chemical BiologyKolkata, India; ^2^Academy of Scientific and Innovative ResearchKolkata, India

**Keywords:** body habitats, host genetics, lifestyle, disease susceptibility, non-western people, hunter-gatherers, rural community, urban life

## Abstract

One of the fundamental issues in the microbiome research is characterization of the healthy human microbiota. Recent studies have elucidated substantial divergences in the microbiome structure between healthy individuals from different race and ethnicity. This review provides a comprehensive account of such geography, ethnicity or life-style-specific variations in healthy microbiome at five major body habitats—Gut, Oral-cavity, Respiratory Tract, Skin, and Urogenital Tract (UGT). The review focuses on the general trend in the human microbiome evolution—a gradual transition in the gross compositional structure along with a continual decrease in diversity of the microbiome, especially of the gut microbiome, as the human populations passed through three stages of subsistence like foraging, rural farming and industrialized urban western life. In general, gut microbiome of the hunter-gatherer populations is highly abundant with *Prevotella*, Proteobacteria, Spirochaetes, Clostridiales, *Ruminobacter etc*., while those of the urban communities are often enriched in *Bacteroides, Bifidobacterium*, and Firmicutes. The oral and skin microbiome are the next most diverse among different populations, while respiratory tract and UGT microbiome show lesser variations. Higher microbiome diversity is observed for oral-cavity in hunter-gatherer group with higher prevalence of *Haemophilus* than agricultural group. In case of skin microbiome, rural and urban Chinese populations show variation in abundance of *Trabulsiella* and *Propionibacterium*. On the basis of published data, we have characterized the core microbiota—the set of genera commonly found in all populations, irrespective of their geographic locations, ethnicity or mode of subsistence. We have also identified the major factors responsible for geography-based alterations in microbiota; though it is not yet clear which factor plays a dominant role in shaping the microbiome—nature or nurture, host genetics or his environment. Some of the geographical/racial variations in microbiome structure have been attributed to differences in host genetics and innate/adaptive immunity, while in many other cases, cultural/behavioral features like diet, hygiene, parasitic load, environmental exposure etc. overshadow genetics. The ethnicity or population-specific variations in human microbiome composition, as reviewed in this report, question the universality of the microbiome-based therapeutic strategies and recommend for geographically tailored community-scale approaches to microbiome engineering.

## Introduction

We share our body space with around 100 trillion microorganisms, collectively known as microbiota (Turnbaugh et al., 2007; Consortium, 2012). The growing perception that our genetic landscape is a summation of the genes embedded in our own genome as well as in genomes of our microbiota (the microbiome), and that our metabolic features present an assemblage of human and microbial traits has led to the launching of numerous microbiome projects worldwide. Recent advancement of culture-independent, high throughput next generation sequencing technologies has enhanced our ability to characterize the human microbiome at various states of health and disease (Turnbaugh et al., 2007; Consortium, 2012). Large-scale endeavors such as the Human Microbiome Project (HMP) have been initiated for characterization of healthy human microbiome (Turnbaugh et al., 2007). Studies are being conducted to explore the plausible disease links of microbiome and efforts are being made to understand how microbiome varies with host lifestyle, genetics, age, nutrition, medication, and environment (Turnbaugh et al., 2006; Blaser et al., 2008; Gao et al., 2008; Islami and Kamangar, 2008; Garrett et al., 2010; Tana et al., 2010; Castellarin et al., 2011; Wang et al., 2011; Kostic et al., 2012; Li et al., 2014; Blekhman et al., 2015; O'Keefe et al., 2015; Falony et al., 2016; Goodrich et al., 2016; Zhernakova et al., 2016).

**Figure 1:**
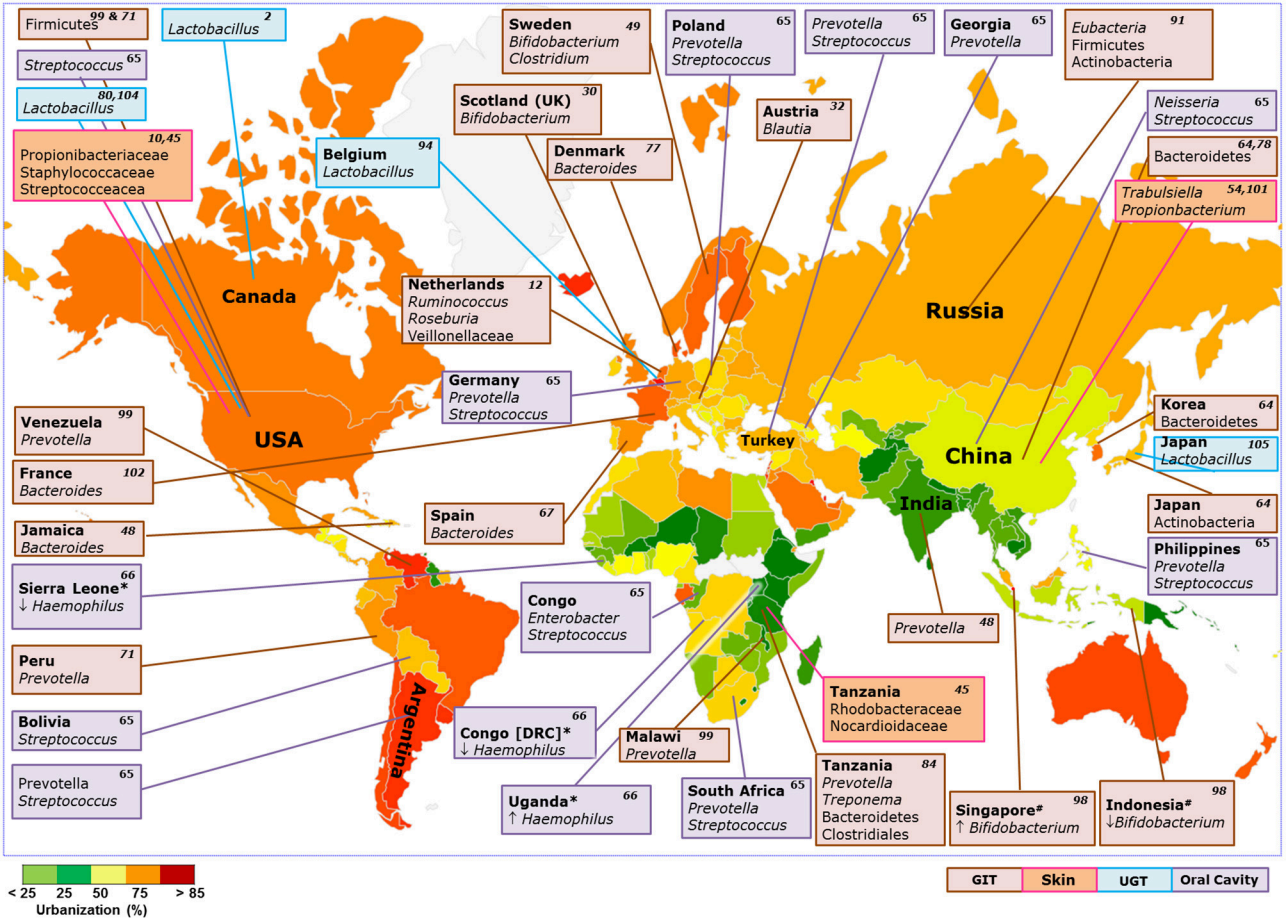
Enriched taxa at various niches of the human body in diverse populations around the world. Box color: body niche; Color in Map: percentage urbanization of countries (http://www.unicef.org/); Up arrow: Dominant abundance of Phylum/Genus compared to respective population; Down arrow: Low abundances of Phylum/Genus/family compared to respective population; ^*^ and ^#^ comparisons between specific countries; Number in respective boxes: Citations.

If we think globally the human microbiome studies are partial, representing for the most part from US, Europe and other so-called WEIRD countries (i.e., Western, Educated, Industrialized, Rich, and Democratic countries) which generally represent urban population (Morton et al., 2015). Only recently, some national and international initiatives have been taken for characterization of human microbiome in diverse ethnic populations and there is a fast growing collection of data describing the microbiome structures in various non-US or non-Western populations (Figure 1) (Moossavi, 2014). These studies have shown significant variations in microbiome composition in healthy individuals from different race and ethnicity categories (Nam et al., 2011; Nasidze et al., 2011; Yap et al., 2011; Yatsunenko et al., 2012; Mason et al., 2013; Li et al., 2014; Schnorr et al., 2014; Leung et al., 2015; Obregon-Tito et al., 2015; Van Treuren et al., 2015; Gomez et al., 2016). Between-group differences in susceptibilities to many health conditions from preterm birth to type 2 diabetes, obesity and even cancer are being linked to microbiome diversity (Peek and Blaser, 2002; Ley et al., 2005; Turnbaugh et al., 2006; Blaser et al., 2008; Gao et al., 2008; Islami and Kamangar, 2008; Garrett et al., 2010; Tana et al., 2010; Castellarin et al., 2011; Wang et al., 2011; Kostic et al., 2012; Blekhman et al., 2015; O'Keefe et al., 2015; Goodrich et al., 2016). It is an established fact that microbiome composition is linked with various diseases, which motivates the scientific community to identify the microbiome based biomarkers for diagnostic and clinical purposes, but population based variation in microbiome composition between healthy individuals makes it difficult. In case of gut microbiome Falony et al. reported a decrease in the number of core genera from 17 to 14 when they analyzed the gut microbiome data from the populations of Papua New Guinea, Peru, and Tanzania with that of a western dataset including data from Flemish and Dutch cohorts, as well as from UK and US populations (Falony et al., 2016). Population based variation in microbiome profile depends on various population based factors for example, Dutch people consume high milk and low antibiotics compared to other populations of Europe (Zhernakova et al., 2016). So population based variation in microbial profile in healthy individuals must be considered to identify the microbiome based biomarkers for particular diseases. In recent times, a number of reviews have been published summarizing microbiome research from various perspectives, but a comprehensive account of the observations made on geography, ethnicity or life-style-specific variations in microbiome composition is long overdue. The present review attempts to address this issue. It will discuss the major findings on cross-population variations in microbiome composition of various biogeographic spaces considering the five major human body habitats—Oral cavity, Respiratory Tract, Gut, Urogenital Tract (UGT) and Skin. The present article also aims to characterize the geographical-core (present in all populations under the study) microbiota at different body habitats of human (Tables S1–S3). Some recent studies attributed variations in microbiome profiles to methodological biases (DNA extraction, primer choice and amplification methods) (Brooks et al., 2015; Walker et al., 2015; Gerasimidis et al., 2016; Vebo et al., 2016). In the present review, most of the included studies are similar in methodology and despite such similarities in methodology, significant variations were observed in the microbiome profiles of different populations. Thus the methodological biases, if any, could not affect the observations made in this review (Table S4). Geography represents an ensemble of genetic, environmental and cultural factors and the degree to which the microbiome is shaped by each of these factors remains debated. It is not clear yet which factor plays a dominant role in shaping the microbiome—nature or nurture, host genetics or his environment, traditions and life-style? The present review has made an attempt toward identification of the factors responsible for geography-based alterations in microbial communities. Possible links between the microbiome structure and the disease susceptibility of the host population has also been discussed. Our work clearly indicates the need for the global association studies between human microbiota and different geographic locations for a proper assessment of the relative importance of diet, ancestry and locations in sculpting the human microbiome architecture.

## Gastrointestinal microbiome

In humans, the gastrointestinal microbiome, especially the gut microbiota (GM), has the largest number of microbes and the greatest variety of species compared to other body habitats. The GM constituents, as predicted from the fecal microbiome, vary substantially across healthy individuals (Consortium, 2012). GM dysbiosis is associated with numerous metabolic and inflammatory disorders like obesity, diabetes, allergy etc. (Adlerberth and Wold, 2009; Armougom et al., 2009; Anderson et al., 2011; Clemente et al., 2012). Cross-population studies on GM usually attempt to address any of the two major issues: (a) influence of host ethnicity and/or life-style on microbiome structure (De Filippo et al., 2010; Yatsunenko et al., 2012; Mardanov et al., 2013; Tyakht et al., 2013; Schnorr et al., 2014; Martinez et al., 2015; Morton et al., 2015; Obregon-Tito et al., 2015; Rampelli et al., 2015; Sankaranarayanan et al., 2015; Gomez et al., 2016) and (b) plausible correlations between variations in GM structure and disease susceptibility (O'Keefe et al., 2007; Ou et al., 2013).

**Figure 2:**
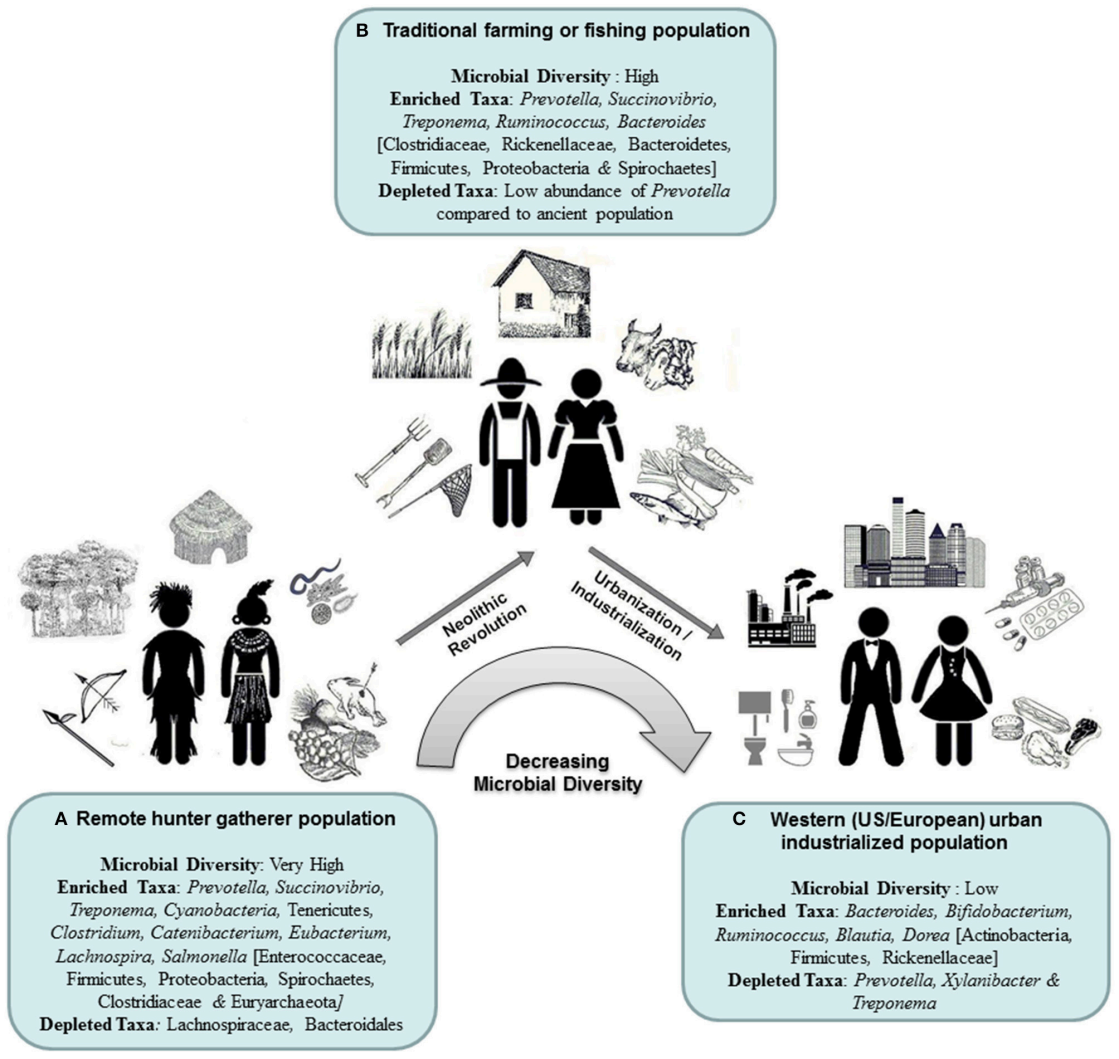
Gradual transition of the gut microbiota composition with changes in the host subsistence strategies.

Table 1 provides the salient features of some cross-population studies that highlighted the influence of host ancestry/life-style on fecal microbiome structure, and Figure 2 depicts the trends in evolution of GM with change in host life-style, as observed in these studies. In most of these studies, investigators compared the GM diversity and composition in two or more representative communities from three distinct subsistence modes: (a) a remote hunter-gatherer population such as the Hadza from Tanzania, Pygmies from Central Africa, the Matses from Peru and Amerindians from Venezuela (O'Keefe et al., 2007; De Filippo et al., 2010; Grzeskowiak et al., 2012; Ou et al., 2013; Schnorr et al., 2014; Morton et al., 2015; Obregon-Tito et al., 2015); (b) a traditional farming or fishing population of localities like the Bantus of Africa, the Tunapuco populations of the Andean highlands or the rural Malawian communities (Gomez et al., 2016); and (c) a representative group of the western (US/European) urban industrialized society (De Filippo et al., 2010; Grzeskowiak et al., 2012; Mardanov et al., 2013; Tyakht et al., 2013; Greenhill et al., 2015; Sankaranarayanan et al., 2015). The hunter-gatherer communities primarily rely on starchy foods like tubers or cassava, plants, nuts, wild game, and honey for sustenance. These remote foragers are known to suffer from multiple infections of gastrointestinal pathogens/parasites, but have little or no access to modern healthcare or medical facilities (Morton et al., 2015; Gomez et al., 2016). The diet of the traditional agriculturalists, on the contrary, is similar to that of people of the Neolithic age, when people switched over from the nomadic lifestyle to settlement in villages, food-crop cultivation and domestication of animals (and fishing and trading at a later stage). People of US metropolitan cities or European countries are typical representatives of the WEIRD societies characterized by urban, industrialized life-style, refined high-protein high-fat diet, improved sanitation and hygiene practices and habitual use of antibiotics and other drugs (De Filippo et al., 2010; Grzeskowiak et al., 2012; Mardanov et al., 2013; Tyakht et al., 2013; Greenhill et al., 2015; Sankaranarayanan et al., 2015). In another study Bengtsson-Palme et al. showed that the gut microbiome varies in abundance of antibiotic resistance genes in their genomes across different geographical populations and acts as a transporter for these genes when people travel from one place to another (Bengtsson-Palme et al., 2015). Some of the cross-population microbiome studies suggested plausible correlations between GM composition and disease susceptibility (Table 1). Fecal metagenomic markers for T2D were found to differ between European and Chinese populations, suggesting that fecal metagenomic predictive tools for T2D should be specific for different geographical populations (Karlsson et al., 2013).

**Table 1:** Geographical/racial variations in gut microbiome composition and diversity.

**S. No**.	**Populations studied**	**Diet/life-style/mode of subsistence**	**Important observations**			**Proposed dominating factors**	**References**
			**Differential composition of GM (phylum/genus/family levels)**	**Predicted enrichment of genes/pathways**	**Any other pertaining remarks**		
1	a) Native Africans (NAs)	Higher dietary intakes of animal proteins, fat and low fiber by AAs than NAs	a) Dominance of *Prevotella* and butyrate-producing groups	a) Genes for hydrogen sulfide production, saccharolytic fermentation, butyrogenesis and methanogenesis	Higher risk of colon cancer in AAs	Diet	O'Keefe et al., 2007; Ou et al., 2013
	b) African Americans (AAs)		b) Higher abundance of *Bacteroides*	b) Genes for secondary bile acid production			
2	a) Children of Burkina Faso (BF) from a rural African village	a) Diet: low in fat and animal protein, rich in starch, fiber, and plant polysaccharides, predominantly vegetarian	a) Higher abundance of Actinobacteria and Bacteroidetes, exclusive presence of *Prevotella, Xylanibacter, Butyrivibrio, and Treponema*	a) Genes for cellulose, xylan hydrolysis and short-chain fatty acids	Higher microbial richness and biodiversity in BF samples than in EU samples	Diet	De Filippo et al., 2010
	b) European children (EU)	b) Typical western diet high in animal protein, sugar, starch, and fat and low in fiber	b) Higher abundance of Firmicutes, Proteobacteria, and Enterobacteriaceae (*Shigella* and *Escherichia*)				
3	a) The Hadza—a hunter- gatherer community of Tanzania, Africa	a) Ancient foraging subsistence Diet: Game meat, honey, baobab, berries and tubers	a) Enriched in *Succinivibrio* sp. *Ruminobacter*, Spirochaetes *(Treponema), Prevotella*, unclassified Bacteroidetes, Firmicutes, Proteobacteria, and Clostridiales	a) Propionate producers	Higher levels of microbial richness and biodiversity in the Hadza than in Italian urban controls	Diet, Sex, Foraging vs. Western lifestyle	Schnorr et al., 2014
	b) Urban Italian adults from Bologna, Italy	b) Western life-style Diet: plant foods, fresh fruit, pasta, bread olive oil; dairy, poultry, fish, and red meat	b) Higher abundance of *Bifidobacterium*, Firmicutes *(Blautia, Ruminococcus, and Faecalibacterium)*	b) Butyrate producers			
4.	a) Amerindian population of Venezuela	a) Ancient subsistence Diet: corn, cassava	a) High abundance of *Prevotella* and Enterococcaceae	460 ECs including those involved in glutamate synthase, alpha-amylase etc. are enriched in non-US populations (a & b) 433 ECs including those involved in vitamin biosynthesis, xenobiotics metabolism, sugar catabolism, bile salt metabolism etc. are enriched in US population 445 ECs are differentially present in Malawian and Amerindian adults Higher representation of Urease (EC3.5.1.5) genes in (a & b)	Significant similarity of the gut microbiome among family members across life-style	Host genetics, age, food habits, geography, differential exposure to pets and livestock etc.	Yatsunenko et al., 2012
	b) Rural Malawian communities	b) Rural agricultural subsistence Diet: maize, fruits, vegetables, ground nut flour etc.	b) High abundance of *Prevotella*				
	c) US metropolitan city dwellers	c) Western, urban, industrialized life-style, protein-rich diet	c) Enriched in *Bacteroides* 56 species-level OTUs are differentially present in Malawian and Amerindian adults 73 OTUs (23 for *Prevotella*) are over represented in non-US adults (a & b)				
5.	a) The Matses from the Peruvian Amazon	a) Isolated hunter-gatherer community Diet: tubers, invasive plantains, fish, game meat	a) Higher abundance of *Succinovibrio, Treponema*, Cyanobacteria, Tenericutes, *Prevotella*, Firmicutes *(Clostridium, Catenibacterium, Eubacterium, Lachnospira etc.)*, Proteobacteria, Spirochaetes and Euryarchaeota	78 KEGG ortholog groups (KOs), mostly associated with metabolism and genetic information processing and 79 ECs (some involved in Tricarboxylic acid cycle) are enriched in Traditional groups (a & b). 20 KOs, mostly associated with membrane transport, and 12 ECs (3 related to Vitamin B1 and B12 biosynthesis) are enriched in urban population (c).	Higher microbial diversity in the Matses and Tunapuco populations than in the Norman population.	Dietary regimes and life-style	Obregon-Tito et al., 2015
	b) Tunapuco populations from the Andean highlands	b) Traditional agriculturalist group Diet: local agricultural products, homegrown small animals	b) Higher abundance of *Succinovibrio, Treponema, Prevotella*, Bacteroidetes, Proteobacteria and Spirochaetes.				
	c) Residents of Norman, Oklahoma, US.	c) Urban-industrialized Western society Diet: canned fruits and vegetables, bread, dairy products, prepackaged western meals	c) Enriched in Actinobacteria *(Bifidobacterium), Bacteroides* and Firmicutes *(Ruminococcus, Blautia, Dorea)*				
6.	a) Pygmy hunter-gatherers	a) Ancient foraging subsistence Diet: cassava, nuts occasional game meat	a) Higher frequencies of Proteobacteria, especially of *Succinivibrio* and *Salmonella*, depleted in Lachnospiraceae family	Only one pathway associated with bacterial invasion of epithelial cells, has been reported to differ significantly across all subsistence types, with the highest relative abundance in the hunter-gatherers and lowest in the farmers	Significant correlation of the microbiome diversity and composition with presence of the gut protozoa *Entamoeba* and the mode of subsistence	Parasitic load, dietary regime, subsistence mode	Morton et al., 2015
	b) Bantu farming populations	b) Rural agricultural subsistence Diet: locally grown cereals, vegetables, meat	b) Higher abundance of Firmicutes, especially of *Ruminococcus, Treponema*				
	c) Bantu fishing populations All three populations were from Southwest Cameroon, Africa	c) Fishing population Diet: cassava, fish, meat, yogurt	c) Enriched in *Bifidobacteria*, Bacteroidales, depleted in *Ruminococcus*				
7.	a) BaAka pygmies from the Central African Republic.	a) Ancient hunter-gatherer subsistence, no exposure to antibiotics or modern therapeutics Diet: wild game, fish, fibrous leaves, nuts and fruits.	a) High abundance of *Prevotella*, Clostridiaceae and *Treponema*, depleted in Bacteroidales	a) 14 pathways including those involved in pathogenicity, peptidoglycan biosynthesis, purine/pyrimidine metabolism etc. Increased abundance of virulence, amino acid, lipid and vitamin metabolism pathways	A gradual change in the microbial profiles from the BaAka → the Bantu → US Americans—consistent with their degree of traditional lifestyle	Diet, life-style, parasitic load, exposure to modern therapeutics	Gomez et al., 2016
	b) Bantu population from the Central African Republic.	b) Intermediate abundance of *Prevotella*, Clostridiaceae, and *Treponema*, relatively enriched in Rickenellaceae and *Bacteroides*. Bantu gut microbiome is dominated b*y* Firmicutes	b) Traditional agriculturist group, partial exposure to western life-style and modern therapeutics Diet: flour-like products, goat meat	b) 22 pathways including transporters, secretion system, signal transduction mechanism etc. Increased abundance of carbohydrate and xenobiotics metabolism pathways			
	c) US Americans (from HMP project)	c) Highly enriched in Rickenellaceae and *Bacteroides*	c) Typical modern western life-style and diet	c) 36 pathways including carbohydrate metabolism, xenobiotics metabolism, amino/nucleotide sugar metabolism etc.			

*EC, Enzyme commission numbers*.

All these studies revealed some general trends in evolution of human GM with gradual transition in host subsistence pattern (Figure 2). The most apparent trend is significantly higher microbial diversity in guts of foraging people and its gradual reduction with westernization in the host community (Schnorr et al., 2014). For instance, the microbiome profiles of the agriculturalist Bantu population exhibit an intermediate state between the microbiomes of Pygmy hunter-gatherers and those of US individuals (Obregon-Tito et al., 2015; Gomez et al., 2016). It has been proposed that the high taxonomic diversity in the wandering foragers might have endowed their gastrointestinal ecosystem with greater stability and functional flexibility, enabling them to withstand the perpetual presence of pathogens or parasites and to respond to fluctuations in diet due to unpredictable and seasonally dependent food supply (Morton et al., 2015).

Divergence in the GM composition, especially the *Prevotella-Bacteroides* trade-off between the hunter-gatherers and western people is often attributed to their dietary specialization (O'Keefe et al., 2007; Ou et al., 2013; Gomez et al., 2016). *Prevotella* is thought to enhance the ability to digest and extract valuable nutrition from fibrous plant foods and *Treponema* also might be advantageous to nutritional acquisition of traditional people because of its fibrinolytic specializations (Obregon-Tito et al., 2015). As compared to foraging populations, the agriculturalists and urban industrialized people exhibit an appreciable enrichment in carbohydrate- and xenobiotic-processing presumably due to their access to more digestible sugars and therapeutic drugs (Gomez et al., 2016).

The role of host genetics in shaping the GM is not very clear yet (Goodrich et al., 2016), though there were some reports on ethnicity or geography-specific variations in GM (Yatsunenko et al., 2012). Yatsunenko et al. noticed a basal level of influence of family and shared environment on microbiome structure, which was found to be fairly consistent across US, Malawian and Amerindian communities (Yatsunenko et al., 2012). Analysis of fecal microbiota of infants, genetically at high risk for Type I Diabetes at six clinical centers in Europe (Finland, Sweden, and Germany) and the U.S. (Colorado, Washington state, and Georgia/Florida) revealed significant association of geographical origin with the diversity and the relative abundance of different bacterial genera, especially of *Bifidobacterium, Veillonella, Faecalibacterium, Streptococcus*, and *Akkermansia* in GM (Kemppainen et al., 2015). The study found significantly lower diversity in the fecal microbiota of Finland and Colorado babies, which was in good agreement with an earlier report on reduction in GM diversity in infants from northern European countries as compared to southern or central European infants (e.g., Sweden vs. Spain and Finland vs. Germany), irrespective of the delivery mode, breast-feeding and antibiotics exposure (Fallani et al., 2010). Geographical and population-specific factors might also have influenced IBD associated changes in gut ecosystems, as indicated by increase in abundance of Firmicutes in GM of IBD patients in European (Germany and Lithuania) population but not in South Asian (Indian) population (Rehman et al., 2016).

However, in many cases, the mode of subsistence exhibited specific microbiome signatures that seemed to overrule ethnic background. For instance, the Bantu-farming and Bantu-fishing communities though sharing the same genetic ancestry, show larger differences in gut microbiome profiles between themselves than between the Pygmies and Bantu-farming population (Morton et al., 2015). In the principal component analysis of genus-level taxa abundance profiles of GM, a clear separation was observed between three subsistence groups—the hunter-gatherer populations (Hadza and Matses from Africa and South America) the rural agriculturalists (Tunapuco, Malawi, and Venezuela from Africa and South America) and the urban industrial populations (from Europe and North America), which clearly indicated that the influence of diet/subsistence on GM may overrule the host ancestry and geographic origin (Obregon-Tito et al., 2015). Dominance of diet and other life-style factors over genetics/ethnicity is also apparent from the compositional differences in gut microbiome between Hadza men and women—significantly higher abundance of *Treponema* in women and increased *Eubacterium* and *Blautia* in men—which may be attributed to the sexual division of labor and sex differences in dietary regimes (Schnorr et al., 2014). The effect of diet on gut microbiome is also apparent from the observation that porphyranase and agarase genes are specific to Japanese gut microbiome and absent from that of western population. It was proposed that these genes might have been acquired by gut bacteria of Japanese from marine bacteria through seaweed diet (Hehemann et al., 2010). A similarity in the gut microbiome composition (dominance of Bacteroidetes and Firmicutes) was observed between humans and domestic cattle and chimpanzees living within the same geographical location (Ellis et al., 2013).

The parasite burden in gut could also be a governing factor for gut ecosystem diversity in the Pygmy hunter-gatherers, Bantu farming and Bantu fishing populations, suggesting potential important interactions between the host immune system, gut microbiome, and gut parasites (Morton et al., 2015). Abundance of some predicted pathways derived from gut microbiome of entamoeba positive and negative individuals were found to be significantly (*P* < 0.05) different, for instance, pathways involved in *biosynthesis of antibiotic tetracycline* and *yeast MAPK signaling pathways* were found in higher abundance in gut microbiome of the BaAka pygmies, who often suffer from multiple infections of pathogenic gastrointestinal parasites (entamoeba) and gut microbiome of entamoeba negative individuals have the higher representation of *cellular antigens pathways* compared to entamoeba positive individuals. These observations advocate for the role of parasitism in shaping the gut microbiota (Morton et al., 2015).

There are many other studies demonstrating geography or ethnicity-specific divergences in GM composition (Figure 1). For instance, American community, both Japanese and Korean communities and Chinese community showed high abundances of Firmicutes, Actinobacteria, and Bacteroidetes, respectively in their GM (Nam et al., 2011). At the genus levels, Japanese (Nishijima et al., 2016) showed high abundances of *Bifidobacterium* and *Clostridium*, Chinese of *Bacteroides* and Korean of *Prevotella* and *Faecalibacterium* in their GM. Nishijima et al., showed dominance of *Prevotella* in Malawi, Venezuela, and Peru; *Bacteroides* in USA, China, Denmark, Spain, and France; *Eubacterium* in Russia; *Clostridium* in Sweden and *Blautia* in Austria (Nam et al., 2011; Nishijima et al., 2016). *Bacteroides* genus dominated in American and Jamaican populations while *Prevotella* genus dominated in Indian population (Kao et al., 2016). *Ruminococcus, Roseburia*, Veillonellaceae dominated in gut microbiome of healthy individuals from the Netherlands (Bonder et al., 2016). Three robust clusters (enterotypes) based on gut microbiome composition were identified by Arumugam et al. but populations specific variations were not well explored (Arumugam et al., 2011).

## Oral microbiome

Microbes that colonize at several niches within our oral cavity after birth and form a stable ecosystem are collectively called oral microbiome. Diseases like periodontitis, teeth reduction, caries and even cancer are known to be associated with imbalance in oral microbiome composition. The fact that susceptibility to these diseases often shows ethnic biases, has inspired the investigators to explore the geographical variations in oral microbiome and its potential impact in oral health. Blekhman et al. observed a link between variation of microbiome composition in oral cavity and host genetic variation when different populations (African vs. American, African vs. European, African vs. Asian, American vs. European, and American vs. Asian) were compared by considering the F_ST_ (Fixation Index) values. Similarly, Li et al. (2014) also showed that saliva microbiome of genetically different populations from Alaska, Germany and Africa were significantly different in alpha (within sample) and beta (between samples) diversity for microbiome composition in saliva. Mason et al. compared the oral microbiome composition between the healthy populations from major races/ethnic groups residing in the United States namely non-Hispanic blacks, non-Hispanic whites, Chinese and Latinos and found that 33 of 77 genera significantly differ in abundance between these four populations (Mason et al., 2013). The study demonstrated ethnicity-specific clustering of microbial communities in saliva and subgingival biofilms and using a machine-learning classifier, it had been possible to identify an individual's ethnicity from sub-gingival microbial signatures with a 100% sensitivity and 74% specificity in African American, 67 and 80% in Latinos and 50 and 91% in Caucasians (Mason et al., 2013). As African Americans and Caucasians share similar environmental factors including food, nutrition, and lifestyle over several generations, it was suggested that the distinct composition of their oral microbiome could be due to the variations in tooth and root morphologies across different ethnic populations as well as to their innate immune responses to infectious agents (Lavelle, 1970, 1971; Cruz et al., 2009; Dewhirst et al., 2010; Mason et al., 2013; Wade, 2013).

These observations contradicted an earlier study by Nasidze et al., who analyzed 16S rRNA sequences from saliva samples from 120 healthy individuals (10 individuals from each of 12 worldwide geographic locations), but did not find any definite correlation of the compositional (presence/absence) variation in saliva microbiota with geographical distances between locations, though abundances of certain genera were significantly different in specific locations (Nasidze et al., 2009). For instance, *Enterobacter* accounted for 28% of total composition in Congo population but was completely absent in samples from California, China, Germany, Poland, and Turkey. California and the Congo showed the largest differences between individuals, while Georgia and Turkey showed the smallest differences between individuals (Nasidze et al., 2009).

A comparative study on salivary microbiome composition in Alaskans, Germans, and Africans revealed more similarities between native Alaskans and Germans than between either group and Africans both at the genus and OTU levels (Li et al., 2014). Abundance of the Firmicutes was highest in Alaskans and Germans, while in Africans, Proteobacteria was the most abundant phylum. Six common genera—*Neisseria, Campylobacter, Granulicatella, Megasphaera, Selenomonas*, and *Actinomyces*-were shared by both Alaskans and Africans but only three genera namely *Actinobacillus, Aggregatibacter*, and *Capnocytophaga* were shared by Germans and Africans (Li et al., 2014). Beta diversity was highest in Africans but alpha diversity in Germans (Li et al., 2014). Four Alaskan groups, located in different regions of Alaska and habituated to distinct diets, did not reveal any significant differences in their saliva microbiome composition. But substantial differences could be observed in the microbiome diversity among three African groups (Nasidze et al., 2011; Li et al., 2014). The Batwa Pygmies, a former hunter-gatherer group from Uganda, showed higher diversity in saliva microbiome than that in the agricultural groups from Sierra Leone and the Democratic Republic of Congo, which may be attributed to the ancient subsistence pattern and protein-rich diet of the pygmies (Nasidze et al., 2011; Li et al., 2014). People of Sierra Leone and Congo, who are geographically distant but have similar life style and diet, showed a higher degree of similarity to each other than with the Batwa. The Batwa population had low incidence of dental caries, presumably due to higher occurrences of *Haemophilus*, which is known to play an important role in pH homeostasis in oral cavity (Nasidze et al., 2011; Li et al., 2014).

## Respiratory tract microbiome

The human respiratory tract is subdivided physiologically into two parts i.e., Upper Respiratory Tract (URT) composed of oropharynx, nasopharynx and nasal cavity and Lower Respiratory Tract (LRT) containing lungs. LRT were traditionally assumed to be sterile, when identification of microorganisms relied on culture based methodologies. But recent advancement of culture independent molecular methodology changed this notion and indicated the presence of microbes in LRT (Charlson et al., 2011). Discovery of human airways microbiome opened the opportunity for understanding the disease onset, exacerbation and progression of chronic respiratory diseases which might be associated with dysbiosis in microbiome (Martin et al., 2015).

Most studies of airways microbiome characterization were focused on URT and few studies of LRT microbiome characterization in healthy human have been restricted to Western European and North American populations (Charlson et al., 2011). The healthy lung microbiome was found indistinguishable from URT except the exclusive presence of *Tropheryma whipplei* in lungs microbiome (Charlson et al., 2011). URT microbiome has been characterized in geographically diverse populations from USA, South Korea (Yi et al., 2014), Netherlands (de Steenhuijsen Piters et al., 2016), and Canada (Stearns et al., 2015) and found mostly similar in all populations. URT microbiota is usually dominated by Firmicutes, Actinobacteria, Proteobacteria, Bacteroidetes, and Fusobacteria phyla, which contain species from *Streptococcus, Neisseria, Gemella, Corynebacterium, Alloiococcus*, and *Haemophilus* genera. The healthy adult lung (LRT) microbiome in US population was dominated by Bacteroidetes, Firmicutes and Proteobacteria phyla, which included the species from *Streptococcus, Veillonella, Prevotella, Pseudomonas, Haemophilus*, and *Neisseria* (Charlson et al., 2011; Morris et al., 2013; Allen et al., 2014; Botero et al., 2014; Bassis et al., 2015; Dickson et al., 2015a,b; Tarabichi et al., 2015). There is no significant difference identified in lung microbiome from eight geographically different cities in USA (Morris et al., 2013).

## Skin microbiome

The skin is the largest body organ of the human, composed of distinct habitats that differ by skin thickness, folds, the density of hair follicles and type of glands. Millions of microbes, mostly commensal, colonize on skin and disruption of this healthy microbiota may cause various diseases (Noble, 1984; Roth and James, 1989; Chiller et al., 2001; Fredricks, 2001; Cogen et al., 2008; Tagami, 2008; Grice and Segre, 2011). Studies suggested that diseases like atopic dermatitis, psoriasis, rosacea, acne etc. are often caused not because of pathogens but due to disruption in normal skin microbiota (Ong et al., 2002; Nomura et al., 2003a,b; de Jongh et al., 2005; Gudjonsson et al., 2009).

Microbial colonization on skin depends on various factors like age, anatomical location, gender, climate, geographical location and exogenous environmental factors (Chen and Tsao, 2013). Understanding of variation in skin microbiome composition in different ethnic or geographical population may explore the reason of variation in susceptibility to certain pathologies or skin disorders in diverse populations. Studies on hand microbiota of women from US and Tanzania showed higher abundances of Propionibacteriaceae, Staphylococcaceae and Streptococceacae families in US population, and that of soil-associated Rhodobacteraceae and Nocardioidaceae in Tanzanian women (Hospodsky et al., 2014). This geographical diversity in skin microbiome might be ascribed to distinct environment and life style in both countries (Hospodsky et al., 2014). US population spends majority of time indoors in contact with dry surfaces, while Tanzanian population performs daily activities in open air in contact with soil, water etc. (Hospodsky et al., 2014).

Comparison of forearm skin specimens from healthy Amerindians in the Venezuelan Amazon and healthy persons in New York and Colorado, US, showed a significant difference in microbiota composition between these two communities (Blaser et al., 2013). The US samples were dominated by *Propionibacterium*. Amerindians were clustered into two groups. One showed bacterial diversity similar to the US community, though it was dominated by *Staphylococcus*. The other group contained a broad range of Proteobacteria with substantially more diversity than the US population and the first group of Amerindians (Blaser et al., 2013). The Amerindians selected for this study represented a population in transition that shifted two or three generations ago from a nomadic hunter-gatherer life-style to permanent homes with access to certain aspects of modern life, yet with a relatively traditional diet (Blaser et al., 2013). However, the factors that distinguished two groups of Amerindian cutaneotypes could not be identified. No definite association of two groups could be found with age, gender, body mass index, relation to drinking water, bathing, use of soap, or other factors (Blaser et al., 2013).

A study on Chinese population showed the considerable variations in skin microbiota between urban and rural population (Ying et al., 2015). *Trabulsiella* was more abundant in urban population than rural, especially on sites including volar forearm, glabella and back of hands (Ying et al., 2015). *Propionibacterium* showed variations in abundance based on skin site and gender between urban and rural dwellers (Ying et al., 2015). In women, *Propionibacterium* on glabella showed the higher abundance in urban than rural population, while *Corynebacterium* exhibited the reverse trend (Ying et al., 2015). The rural adults and elderly people that participated in the study were all agricultural field-workers and hence were exposed to soil, aquatic and other environmental microbial sources that could alter their skin microbiome composition (Ying et al., 2015). On the other hand, most urban subjects had indoor occupations and thus their skin microbiome are predominantly human-derived with little contribution from environmental sources (Ying et al., 2015).

A study on Hong Kong population has implemented the concept of pan microbiome—the total number of microbial species in a specific population (Leung et al., 2015). In this study, the investigators found a steady increase in the size of the pan microbiome, as populations from US, Tanzania and China were included in the dataset, indicating variations in skin microbiome across the countries (Leung et al., 2015).

## Vaginal microbiome

The urogenital tract (UGT) microbiome has been characterized mainly in samples from female subjects derived from vaginal sites. *Lactobacillus* species are known to be the major component of the healthy vaginal microbiota but some studies indicated that *Gardnerella, Atopobium, Prevotella, Pseudomonas*, or *Streptococcus* species are predominant in some healthy women instead of *Lactobacillus* (Hyman et al., 2005; Zhou et al., 2007; Fettweis et al., 2014).

Vaginal microbiome of North American-Asian, North American-white and North American-black women was dominated by *Lactobacillus* (51–96%) but in Belgium population vaginal microbiome was dominated by *Bacteroides* (34%) (Ravel et al., 2011; Verstraelen et al., 2016).

In 2007, a study by Zhou et al. demonstrated significant divergences in vaginal microbiome composition between healthy Caucasian and black women of reproductive age in North America. Microbial communities dominated by *Lactobacillus species* were found to be common in Caucasian women, while the communities dominated by *Atopobium* and a diverse array of phylotypes from the order Clostridiales prevailed in black women (Zhou et al., 2007). A 16S rRNA gene survey of vaginal samples of 396 asymptomatic North American women from four ethnic groups (white, black, Hispanic, and Asian) elucidated existence of five distinct groups of vaginal microbiome profiles: four were dominated by *Lactobacillus crispatus, L. gasseri, L. iners*, or *L. jensenii*, while the fifth contained lower proportions of lactic acid bacteria and higher proportions of strictly anaerobic organisms (Ravel et al., 2011). The proportions of each community group varied considerably among the four ethnic groups, with *Lactobacillus* prevailing among the Asian and white women and anaerobic species being abundant in black and Hispanic women (Ravel et al., 2011). A similar type of study on apparently healthy Japanese women in Tokyo, and White and Black women from North America showed incidences of vaginal communities with several non-*Lactobacillus* species gradually increase from White to Japanese to Black populations (Zhou et al., 2010).

Analysis of vaginal microbiome composition in healthy and diseased African-American and European-American women by Fettweis et al. reconfirmed that European-American vaginal microbiome have the low bacterial diversity dominated by *Lactobacillus* species, while vaginal microbiome of African-American women is more diverse in nature and dominated by *Gardnerella vaginalis* and the uncultivated bacterial vaginosis-associated bacterium-1 (BVAB1) (Fettweis et al., 2014). These observations comply with higher occurrences of bacterial vaginosis among African-American women than among European-American population. Moreover, the prevalence of various bacterial taxa that are known to be associated with microbial invasion of the amniotic cavity and preterm birth such as *Mycoplasma, Gardnerella, Prevotella, Sneathia* etc. also differed between the two ethnic groups (Fettweis et al., 2014). Almost similar type of divergences in vaginal microbiome has also been observed between Belgian (Verstraelen et al., 2016) and Canadian women (Albert et al., 2015), with *L. crispatus. L. iners*, and *Prevotella* prevailing in Belgian population and *L. iners, L. jensenii*, and *G. vaginalis* in Canadian population.

Whether the variation along ethnic lines is a reflection of genetics or environment remains a matter of conjecture. Zhou et al. proposed that host genetic factors, including the innate and adaptive immune systems, may be more important in shaping the composition of vaginal microbiota than the cultural and behavioral differences among ethnic groups such as multiple sex partners, douching, and the use of contraception devices (Zhou et al., 2007). The vaginal bacterial communities of Japanese women resemble those of women in other racial groups (white and black women from North America) (Zhou et al., 2010). But in other ethnic populations (African American and European) Fettweis et al. found significant correlation not only of ethnicity, but also of no-pregnancy and less-alcohol use with the higher relative abundance of bacterial vaginosis associated species (Fettweis et al., 2014).

## Geographically conserved core microbiome

**Figure 3:**
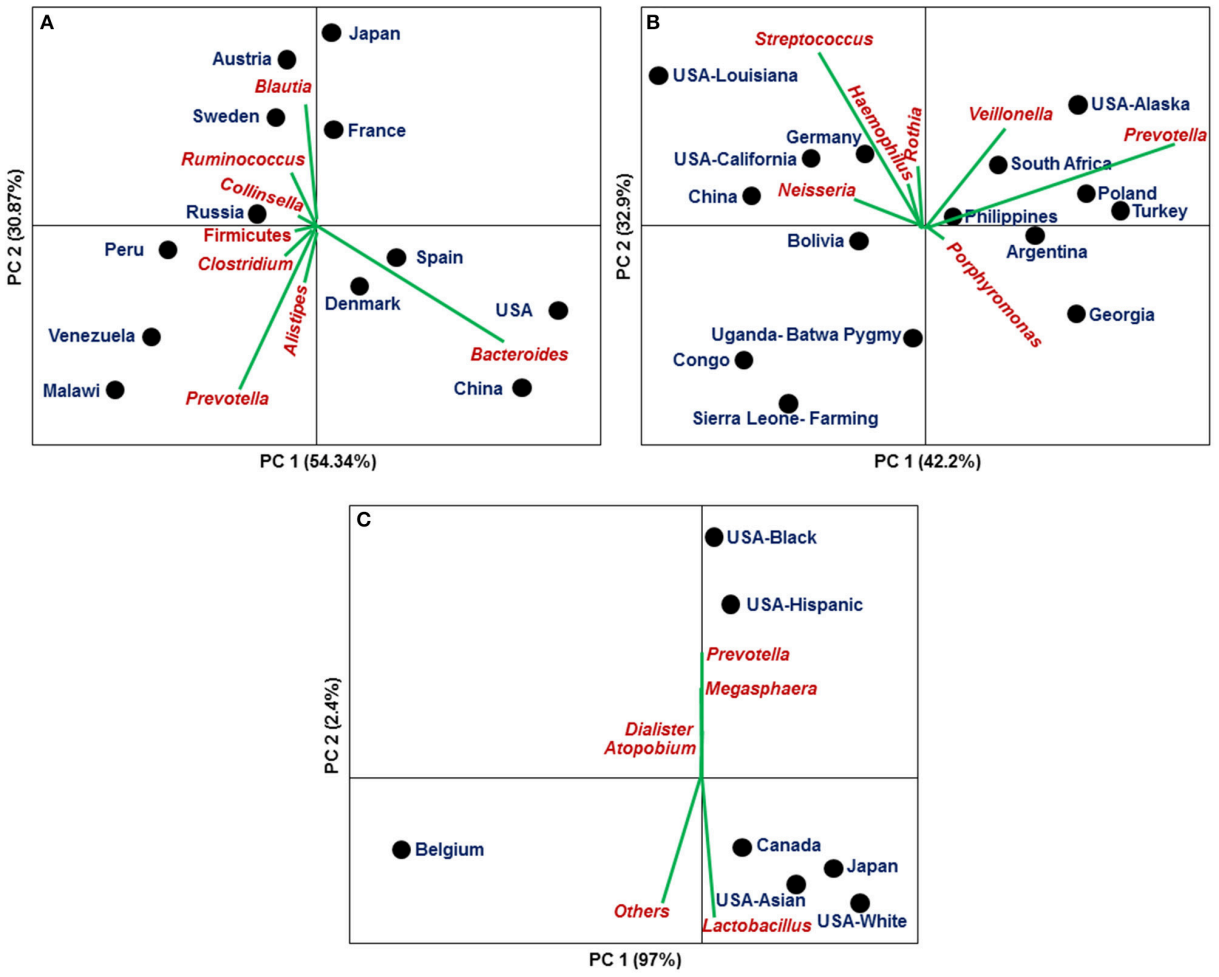
Principal Component Analysis based on relative abundances of core microbiota among different countries/populations derived from a specific body niche (population wise data shown in Tables S1–S3): **(A)** Gut **(B)** Oral cavity **(C)** Vagina.

Apart from a detail discussion of geography, dietary habits, ethnicity or local environment (rural/urban) specific variations in human microbiome, the present article also aims to characterize the geographical-core (present in all populations under the study) microbiota at different body habitats of human. This is totally based on the published data on relative abundance of microbial communities at distinct body niches in healthy human subjects (Figure 3, Tables S1–S3). The core microbiota of a specific body site of human refers to the set of the genera, which are commonly found in that specific body site of all populations studied so far, irrespective of their geographic locations, ethnic background or places of dwelling. In 2016, Falony et al. also identified a core microbiome (based on individuals) but in this present analysis we considered the populations instead of individuals for estimating the geographical-core microbiome (Figure 3, Tables S1–S3) (Falony et al., 2016). Size of the geographical core indicates the effect of geographical factors on microbiome composition that might be useful to understand the geographical exclusiveness of a specific microbial community.

In case of stool derived microbiome, 25 genera are found to be common in all populations from 12 different countries, though the abundances of these genera have been found to vary substantially across populations (Figure 3A, Table S1) (Qin et al., 2010, 2012; Yatsunenko et al., 2012; Karlsson et al., 2013; Tyakht et al., 2013; Zeller et al., 2014; Feng et al., 2015; Obregon-Tito et al., 2015; Nishijima et al., 2016). For instance, *Bacteroides* are dominant in people from USA (38%), China (39%), Spain (23%), and Denmark (20%) compared to Malawi (3%), Peru (1%), Russia (8%), Venezuela (3%), and Sweden (8%), while *Prevotella* is enriched in Malawi (32%), Peru (14%), and Venezuela (24%) as compared to USA (4%) and Canada (4%) (Qin et al., 2010, 2012; Yatsunenko et al., 2012; Karlsson et al., 2013; Tyakht et al., 2013; Zeller et al., 2014; Feng et al., 2015; Obregon-Tito et al., 2015; Nishijima et al., 2016).

Salivary microbiome as a part of the oral microbiome are characterized in 15 populations from 13 geographically diverse countries, but only 15 genera are commonly present in all 15 populations (Nasidze et al., 2009, 2011; Li et al., 2014). In Congo, Sierra Leone, Uganda and Bolivia population, core microbiota covers only 41, 33, 58, and 65% of total salivary microbiota, respectively (Figure 3B, Table S2). This variation in relative abundances of core microbial communities indicate the effect of population specific factors like diet, genetics, life style, use of antibiotics, occupation behaviors etc.

Different number of genera has been found in vaginal microbiome of various individuals from 7 different populations from 4 countries (Belgian, US-Asian, US-white, US-Hispanic, US-black, Canadian, and Japanese). Out of all characterized genera only 5 genera (>0.1%) are shared among all seven populations (Figure 3C, Table S3) (Zhou et al., 2010; Ravel et al., 2011; Albert et al., 2015; Verstraelen et al., 2016). All populations except the Belgians (~16%) are dominated (>60%) by *Lactobacillus*. Four ethnic populations from USA were clustered into two groups (White/Asian and Black/Hispanic) in their vaginal microbiome composition (Figure 3C).

With a view to identify the primary causes of variations in the core microbiome composition (relative abundances) of specific body niches across different populations, we have conducted the Principal Component Analysis (PCA) of the core microbiota composition in stool, saliva and vagina using abundances of core microbiome of 12, 15, and 7 different populations, respectively. Figure 3 depicts the Axis 1 vs. Axis 2 plot of PCA of the stool (Figure 3A), saliva (Figure 3B) and vaginal (Figure 3C) microbiome of different populations, where the directions of increase of various bacterial genera are indicated by specific arrows, which indicates the population specific dominance of a specific microbial community (Figure 3). These observations clearly indicate that though there exists a core microbiome in each specific body-niche, distinct bacterial genus/genera dominate in healthy individuals of different geographical and/or ethnic populations. Figure 3 showed that most of the variance is accounted by first two principal components in vaginal microbiome (~99%), stool (~85%), and saliva (~75%). All geographical populations are grouped in different clusters on respective PCA plots for example, gut microbiome is grouped in 3 main clusters as shown in Figure 3A (Cluster 1 [*Bacteroides* dominance]: Denmark, Spain, USA and China; Cluster 2 [*Prevotella* dominance]: Peru, Venezuela, Russia and Malawi; Cluster 3 [*Ruminococcus* and *Blautia* dominance]: Austria, Sweden, Japan, and France); saliva microbiome also clustered into 3 groups as shown in Figure 3B which comprise differential dominance of microbial genera for example, Cluster 1: *Streptococcus, Neisseria, and Haemophilus* (USA-Louisiana, USA-California, Germany, and China); Cluster 2: *Prevotella* and *Veillonella* (USA-Alaska, South Africa, Poland, Turkey, Philippines, and Argentina); the vaginal microbiome is clustered into 2 groups (Cluster 1 [*Lactobacillus* dominance]: USA-Black and USA-Hispanic; Cluster 2 [*Prevotella* dominance]: Canada, Japan, USA-Asian and USA-White) but Belgium population is segregated from both groups (Figure 3C).

## Summary

Studies conducted on human microbiome so far revealed some general trends observed in variations in micro-ecology at distinct body habitats across various countries/populations around the world (Figure 1). It appears that there had been a gradual transition in the gross compositional structure and decrease in diversity of the human microbiome, especially in the gut microbiota, as the human populations passed through three stages of subsistence—foraging, rural farming and industrialized urban life. Higher microbial diversity in nomadic hunter-gatherers had probably endowed them with greater stability and flexibility, enabling them to cope with challenging ecology. Changes in human diet, lifestyle and traditions brought about by the Neolithic revolution have been reflected in the microbiota, especially in the gut microbiome of the traditional agriculturalists. With time, urbanization and industrialization have gradually led to modern people of developed countries, adapted to indoor-based secured life-style, consumption of refined high protein foods, improved sanitation, less exposure to soil, forest or domestic animals and habitual use of antibiotics—all having a dramatic impact on the functional role of the western microbiome.

Much of the mutualistic functions of the ancestral human microbiome that could have facilitated our primitive forerunners to fight and survive against adverse environment are no more required and a substantial part of the ancient microbial ensemble that our ancestors shared has probably been lost through the adoption of modern urban, industrial, western lifestyle. For instance, exclusive presence of *Treponema* in the gut of all traditional people studied to date (and also in non-human primates), but not in Western urban populations suggested that *Treponema* might be one of the symbionts lost in present-day urban-industrialized societies. This microbial simplification has probably left us with depleted microbiome, deficient in beneficial microbes that once might have played important metabolic roles in human health and this, perhaps, explains the rise of several “diseases of civilization” like allergies, diabetes, asthma, obesity, inflammatory bowel disease and so on.

The debate on “nature” vs. “nurture” remains to be resolved. Some of the geographical/racial variations in microbiome structure have been attributed to differences in host genetics and innate/adaptive immunity, while in many other cases, cultural/behavioral features like diet, hygiene, environmental exposure etc. overshadow genetics. Especially the diet and subsistence mode of the host population often inscribe their signatures in the gut microbiome diversity and composition, irrespective of the geographic origin, ethnicity or local environment of host population. The fact that despite large geographic and ethnic divergences, the hunter-gatherer populations like the Hadza from Africa and the Matses from South America or the rural agriculturalists like the Tunapuco from Africa and the Malawi and Venezuela from South America exhibit similarity in their gut microbiome structure advocates for the dominance of nurture over nature (Nam et al., 2011; Schnorr et al., 2014). On the contrary, host genetics and immunity are considered to be the major factors in shaping the UGT (vaginal) bacterial profiles, though hygiene, mode of pregnancy or alcohol addiction seems to have substantial influences (Zhou et al., 2010). Taken together, these conjectures motivate the need for larger association studies to assess the relative importance of host ancestry, diet, hygiene and other life-style factors in sculpting the human microbiome architecture.

## Conclusion

Reports on ethnicity or population-specific variations in human microbiome composition question the universality of the microbiome-based therapeutic strategies and recommend for geographically tailored community-scale approaches to microbiome engineering. Generic microbiome manipulations, designed on the basis of studies on WEIRD societies, may have unintended, and even adverse consequences in non-western populations. However, designing a geographically tailored therapeutic approach would need an in-depth understanding of how population and environmental parameters can affect the microbial communities and their metabolic potentials, which, we hope, may be attained in near future through construction of pan microbiome of human populations around the globe.

## Author contributions

VG developed the first draft of this review. SP provided suggestions for expansion and new direction and final editing. CD guided, coordinated the study and revised the manuscript critically for important intellectual content. All authors read and approved the final manuscript.

### Conflict of interest statement

The authors declare that the research was conducted in the absence of any commercial or financial relationships that could be construed as a potential conflict of interest.

## Abbreviations

UGT: Urogenital tract
HMP: Human Microbiome Project
WEIRD: Western, Educated, Industrialized, Rich and Democratic countries
GM: Gut Microbiota
IBD: Inflammatory bowel Disease
AAs: African Americans
NAs: Native Africans
T2D: Type-2 Diabetes
NAFLD: Non-alcoholic fatty liver disease
CCHC: Cameron County Hispanic Cohort
OTU: Operational Taxonomic Unit
URT: Upper respiratory Tract
LRT: Lower Respiratory Tract
BVAB1: Bacterial Vaginosis-Associated Bacterium-1
GIT: Gastrointestinal Tract
PCA: Principal Component Analysis.

## References

[B1] Adlerberth I. Wold A. E. (2009). Establishment of the gut microbiota in Western infants. Acta Paediatr. 98, 229–238. 10.1111/j.1651-2227.2008.01060.x 19143664

[B2] Albert A. Y. Chaban B. Wagner E. C. Schellenberg J. J. Links M. G. van Schalkwyk J.. (2015). A study of the vaginal microbiome in healthy canadian women utilizing cpn60-based molecular profiling reveals distinct gardnerella subgroup community state types. PLoS ONE 10:e0135620. 10.1371/journal.pone.0135620 26266808 PMC4534464

[B3] Allen E. K. Koeppel A. F. Hendley J. O. Turner S. D. Winther B. Sale M. M. (2014). Characterization of the nasopharyngeal microbiota in health and during rhinovirus challenge. Microbiome 2:22. 10.1186/2049-2618-2-22 25028608 PMC4098959

[B4] Anderson C. A. Boucher G. Lees C. W. Franke A. D'Amato M. Taylor K. D.. (2011). Meta-analysis identifies 29 additional ulcerative colitis risk loci, increasing the number of confirmed associations to 47. Nat. Genet. 43, 246–252. 10.1038/ng.764 21297633 PMC3084597

[B5] Armougom F. Henry M. Vialettes B. Raccah D. Raoult D. (2009). Monitoring bacterial community of human gut microbiota reveals an increase in Lactobacillus in obese patients and Methanogens in anorexic patients. PLoS ONE 4:e7125. 10.1371/journal.pone.0007125 19774074 PMC2742902

[B6] Arumugam M. Raes J. Pelletier E. Le Paslier D. Yamada T. Mende D. R.. (2011). Enterotypes of the human gut microbiome. Nature 473, 174–180. 10.1038/nature09944 21508958 PMC3728647

[B7] Bassis C. M. Erb-Downward J. R. Dickson R. P. Freeman C. M. Schmidt T. M. Young V. B.. (2015). Analysis of the upper respiratory tract microbiotas as the source of the lung and gastric microbiotas in healthy individuals. MBio 6:e00037. 10.1128/mBio.00037-15 25736890 PMC4358017

[B8] Bengtsson-Palme J. Angelin M. Huss M. Kjellqvist S. Kristiansson E. Palmgren H.. (2015). The human gut microbiome as a transporter of antibiotic resistance genes between continents. Antimicrob. Agents Chemother. 59, 6551–6560. 10.1128/AAC.00933-15 26259788 PMC4576037

[B9] Blaser M. J. Chen Y. Reibman J. (2008). Does *Helicobacter pylori* protect against asthma and allergy? Gut 57, 561–567. 10.1136/gut.2007.133462 18194986 PMC3888205

[B10] Blaser M. J. Dominguez-Bello M. G. Contreras M. Magris M. Hidalgo G. Estrada I.. (2013). Distinct cutaneous bacterial assemblages in a sampling of South American Amerindians and US residents. ISME J. 7, 85–95. 10.1038/ismej.2012.81 22895161 PMC3526177

[B11] Blekhman R. Goodrich J. K. Huang K. Sun Q. Bukowski R. Bell J. T.. (2015). Host genetic variation impacts microbiome composition across human body sites. Genome Biol. 16:191. 10.1186/s13059-015-0759-1 26374288 PMC4570153

[B12] Bonder M. J. Tigchelaar E. F. Cai X. Trynka G. Cenit M. C. Hrdlickova B.. (2016). The influence of a short-term gluten-free diet on the human gut microbiome. Genome Med. 8:45. 10.1186/s13073-016-0295-y 27102333 PMC4841035

[B13] Botero L. E. Delgado-Serrano L. Cepeda M. L. Bustos J. R. Anzola J. M. Del Portillo P.. (2014). Respiratory tract clinical sample selection for microbiota analysis in patients with pulmonary tuberculosis. Microbiome 2:29. 10.1186/2049-2618-2-29 25225609 PMC4164332

[B14] Brooks J. P. Edwards D. J. Harwich M. D. Rivera M. C. Jr. Fettweis J. M. Serrano M. G.. (2015). The truth about metagenomics. quantifying and counteracting bias in 16S rRNA studies. BMC Microbiol. 15:66. 10.1186/s12866-015-0351-6 25880246 PMC4433096

[B15] Castellarin M. Warren R. L. Freeman J. D. Dreolini L. Krzywinski M. Strauss J.. (2011). Fusobacterium nucleatum infection is prevalent in human colorectal carcinoma. Genome Res. 22, 299–306. 10.1101/gr.126516.111 22009989 PMC3266037

[B16] Charlson E. S. Bittinger K. Haas A. R. Fitzgerald A. S. Frank I. Yadav A.. (2011). Topographical continuity of bacterial populations in the healthy human respiratory tract. Am. J. Respir. Crit. Care Med. 184, 957–963. 10.1164/rccm.201104-0655OC 21680950 PMC3208663

[B17] Chen Y. E. Tsao H. (2013). The skin microbiome: current perspectives and future challenges. J. Am. Acad. Dermatol. 69, 143–155. 10.1016/j.jaad.2013.01.016 23489584 PMC3686918

[B18] Chiller K. Selkin B. A. Murakawa G. J. (2001). Skin microflora and bacterial infections of the skin. J. Investig. Dermatol. Symp. Proc. 6, 170–174. 10.1046/j.0022-202x.2001.00043.x 11924823

[B19] Clemente J. C. Ursell L. K. Parfrey L. W. Knight R. (2012). The impact of the gut microbiota on human health: an integrative view. Cell 148, 1258–1270. 10.1016/j.cell.2012.01.035 22424233 PMC5050011

[B20] Cogen A. L. Nizet V. Gallo R. L. (2008). Skin microbiota: a source of disease or defence? Br. J. Dermatol. 158, 442–455. 10.1111/j.1365-2133.2008.08437.x 18275522 PMC2746716

[B21] Consortium T. H. M. P. (2012). Structure, function and diversity of the healthy human microbiome. Nature 486, 207–214. 10.1038/nature11234 22699609 PMC3564958

[B22] Cruz G. D. Chen Y. Salazar C. R. Le Geros R. Z. (2009). The association of immigration and acculturation attributes with oral health among immigrants in New York City. Am. J. Public Health 99(Suppl. 2), S474–S480. 10.2105/ajph.2008.149799 19443820 PMC4504384

[B23] De Filippo C. Cavalieri D. Di Paola M. Ramazzotti M. Poullet J. B. Massart S.. (2010). Impact of diet in shaping gut microbiota revealed by a comparative study in children from Europe and rural Africa. Proc. Natl. Acad. Sci. U.S.A. 107, 14691–14696. 10.1073/pnas.1005963107 20679230 PMC2930426

[B24] de Jongh G. J. Zeeuwen P. L. Kucharekova M. Pfundt R. van der Valk P. G. Blokx W.. (2005). High expression levels of keratinocyte antimicrobial proteins in psoriasis compared with atopic dermatitis. J. Invest. Dermatol. 125, 1163–1173. 10.1111/j.0022-202X.2005.23935.x 16354186

[B25] de Steenhuijsen Piters W. A. Huijskens E. G. Wyllie A. L. Biesbroek G. van den Bergh M. R. Veenhoven R. H.. (2016). Dysbiosis of upper respiratory tract microbiota in elderly pneumonia patients. ISME J. 10, 97–108. 10.1038/ismej.2015.99 26151645 PMC4681870

[B26] Dewhirst F. E. Chen T. Izard J. Paster B. J. Tanner A. C. Yu W. H.. (2010). The human oral microbiome. J. Bacteriol. 192, 5002–5017. 10.1128/JB.00542-10 20656903 PMC2944498

[B27] Dickson R. P. Erb-Downward J. R. Freeman C. M. McCloskey L. Beck J. M. Huffnagle G. B.. (2015a). Spatial variation in the healthy human lung microbiome and the adapted Island model of lung biogeography. Ann. Am. Thorac. Soc. 12, 821–830. 10.1513/AnnalsATS.201501-029OC 25803243 PMC4590020

[B28] Dickson R. P. Erb-Downward J. R. Martinez F. J. Huffnagle G. B. (2015b). The microbiome and the respiratory tract. Annu. Rev. Physiol. 78, 481–504. 10.1146/annurev-physiol-021115-105238 26527186 PMC4751994

[B29] Ellis R. J. Bruce K. D. Jenkins C. Stothard J. R. Ajarova L. Mugisha L.. (2013). Comparison of the distal gut microbiota from people and animals in Africa. PLoS ONE 8:e54783. 10.1371/journal.pone.0054783 23355898 PMC3552852

[B30] Fallani M. Young D. Scott J. Norin E. Amarri S. Adam R.. (2010). Intestinal microbiota of 6-week-old infants across Europe: geographic influence beyond delivery mode, breast-feeding, and antibiotics. J. Pediatr. Gastroenterol. Nutr. 51, 77–84. 10.1097/MPG.0b013e3181d1b11e 20479681

[B31] Falony G. Joossens M. Vieira-Silva S. Wang J. Darzi Y. Faust K.. (2016). Population-level analysis of gut microbiome variation. Science 352, 560–564. 10.1126/science.aad3503 27126039

[B32] Feng Q. Liang S. Jia H. Stadlmayr A. Tang L. Lan Z.. (2015). Gut microbiome development along the colorectal adenoma-carcinoma sequence. Nat. Commun. 6:6528. 10.1038/ncomms7528 25758642

[B33] Fettweis J. M. Brooks J. P. Serrano M. G. Sheth N. U. Girerd P. H. Edwards D. J.. (2014). Differences in vaginal microbiome in African American women versus women of European ancestry. Microbiology 160, 2272–2282. 10.1099/mic.0.081034-0 25073854 PMC4178329

[B34] Fredricks D. N. (2001). Microbial ecology of human skin in health and disease. J. Investig. Dermatol. Symp. Proc. 6, 167–169. 10.1046/j.0022-202x.2001.00039.x 11924822

[B35] Gao Z. Tseng C. H. Strober B. E. Pei Z. Blaser M. J. (2008). Substantial alterations of the cutaneous bacterial biota in psoriatic lesions. PLoS ONE 3:e2719. 10.1371/journal.pone.0002719 18648509 PMC2447873

[B36] Garrett W. S. Gallini C. A. Yatsunenko T. Michaud M. DuBois A. Delaney M. L.. (2010). Enterobacteriaceae act in concert with the gut microbiota to induce spontaneous and maternally transmitted colitis. Cell Host Microbe 8, 292–300. 10.1016/j.chom.2010.08.004 20833380 PMC2952357

[B37] Gerasimidis K. Bertz M. Quince C. Brunner K. Bruce A. Combet E.. (2016). The effect of DNA extraction methodology on gut microbiota research applications. BMC Res. Notes 9:365. 10.1186/s13104-016-2171-7 27456340 PMC4960752

[B38] Gomez A. Petrzelkova K. J. Burns M. B. Yeoman C. J. Amato K. R. Vlckova K.. (2016). Gut microbiome of coexisting BaAka pygmies and bantu reflects gradients of traditional subsistence patterns. Cell Rep. 14, 2142–2153. 10.1016/j.celrep.2016.02.013 26923597

[B39] Goodrich J. K. Davenport E. R. Beaumont M. Jackson M. A. Knight R. Ober C.. (2016). Genetic determinants of the gut microbiome in UK twins. Cell Host Microbe 19, 731–743. 10.1016/j.chom.2016.04.017 27173935 PMC4915943

[B40] Greenhill A. R. Tsuji H. Ogata K. Natsuhara K. Morita A. Soli K.. (2015). Characterization of the gut microbiota of Papua New Guineans using reverse transcription quantitative PCR. PLoS ONE 10:e0117427. 10.1371/journal.pone.0117427 25658868 PMC4319852

[B41] Grice E. A. Segre J. A. (2011). The skin microbiome. Nat. Rev. Microbiol. 9, 244–253. 10.1038/nrmicro2537 21407241 PMC3535073

[B42] Grzeskowiak L. Collado M. C. Mangani C. Maleta K. Laitinen K. Ashorn P.. (2012). Distinct gut microbiota in southeastern African and northern European infants. J. Pediatr. Gastroenterol. Nutr. 54, 812–816. 10.1097/MPG.0b013e318249039c 22228076

[B43] Gudjonsson J. E. Ding J. Li X. Nair R. P. Tejasvi T. Qin Z. S.. (2009). Global gene expression analysis reveals evidence for decreased lipid biosynthesis and increased innate immunity in uninvolved psoriatic skin. J. Invest. Dermatol. 129, 2795–2804. 10.1038/jid.2009.173 19571819 PMC2783967

[B44] Hehemann J. H. Correc G. Barbeyron T. Helbert W. Czjzek M. Michel G. (2010). Transfer of carbohydrate-active enzymes from marine bacteria to Japanese gut microbiota. Nature 464, 908–912. 10.1038/nature08937 20376150

[B45] Hospodsky D. Pickering A. J. Julian T. R. Miller D. Gorthala S. Boehm A. B.. (2014). Hand bacterial communities vary across two different human populations. Microbiology 160, 1144–1152. 10.1099/mic.0.075390-0 24817404

[B46] Hyman R. W. Fukushima M. Diamond L. Kumm J. Giudice L. C. Davis R. W. (2005). Microbes on the human vaginal epithelium. Proc. Natl. Acad. Sci. U.S.A. 102, 7952–7957. 10.1073/pnas.0503236102 15911771 PMC1142396

[B47] Islami F. Kamangar F. (2008). Helicobacter pylori and esophageal cancer risk: a meta-analysis. Cancer Prev. Res. 1, 329–338. 10.1158/1940-6207.CAPR-08-0109 19138977 PMC3501739

[B48] Kao C. C. Hsu J. W. Dwarkanath P. Karnes J. M. Baker T. M. Bohren K. M.. (2016). Indian women of childbearing age do not metabolically conserve arginine as do American and Jamaican women. J. Nutr. 145, 884–892. 10.3945/jn.114.208231 25833892

[B49] Karlsson F. H. Tremaroli V. Nookaew I. Bergstrom G. Behre C. J. Fagerberg B.. (2013). Gut metagenome in European women with normal, impaired and diabetic glucose control. Nature 498, 99–103. 10.1038/nature12198 23719380

[B50] Kemppainen K. M. Ardissone A. N. Davis-Richardson A. G. Fagen J. R. Gano K. A. Leon-Novelo L. G.. (2015). Early childhood gut microbiomes show strong geographic differences among subjects at high risk for type 1 diabetes. Diabetes Care 38, 329–332. 10.2337/dc14-0850 25519450 PMC4302256

[B51] Kostic A. D. Gevers D. Pedamallu C. S. Michaud M. Duke F. Earl A. M.. (2012). Genomic analysis identifies association of Fusobacterium with colorectal carcinoma. Genome Res. 22, 292–298. 10.1101/gr.126573.111 22009990 PMC3266036

[B52] Lavelle C. L. (1970). Crowding and spacing within the human dental arch of different racial groups. Arch. Oral Biol. 15, 1101–1103. 10.1016/0003-9969(70)90123-8 5275864

[B53] Lavelle C. L. (1971). Mandibular molar tooth configurations in different racial groups. J. Dent. Res. 50:1353. 10.1177/00220345710500054501 5285798

[B54] Leung M. H. Wilkins D. Lee P. K. (2015). Insights into the pan-microbiome: skin microbial communities of Chinese individuals differ from other racial groups. Sci. Rep. 5:11845. 10.1038/srep11845 26177982 PMC4503953

[B55] Ley R. E. Backhed F. Turnbaugh P. Lozupone C. A. Knight R. D. Gordon J. I. (2005). Obesity alters gut microbial ecology. Proc. Natl. Acad. Sci. U.S.A. 102, 11070–11075. 10.1073/pnas.0504978102 16033867 PMC1176910

[B56] Li J. Quinque D. Horz H. P. Li M. Rzhetskaya M. Raff J. A.. (2014). Comparative analysis of the human saliva microbiome from different climate zones: Alaska, Germany, and Africa. BMC Microbiol. 14:316. 10.1186/s12866-014-0316-1 25515234 PMC4272767

[B57] Mardanov A. V. Babykin M. M. Beletsky A. V. Grigoriev A. I. Zinchenko V. V. Kadnikov V. V.. (2013). Metagenomic analysis of the dynamic changes in the gut microbiome of the participants of the MARS-500 experiment, simulating long term space flight. Acta Nat. 5, 116–125. 24303207 PMC3848073

[B58] Martin C. Burgel P. R. Lepage P. Andrejak C. de Blic J. Bourdin A.. (2015). Host-microbe interactions in distal airways: relevance to chronic airway diseases. Eur. Respir. Rev. 24, 78–91. 10.1183/09059180.00011614 25726559 PMC9487770

[B59] Martinez I. Stegen J. C. Maldonado-Gomez M. X. Eren A. M. Siba P. M. Greenhill A. R.. (2015). The gut microbiota of rural papua new guineans. composition, diversity patterns, and ecological processes. Cell Rep. 11, 527–538. 10.1016/j.celrep.2015.03.049 25892234

[B60] Mason M. R. Nagaraja H. N. Camerlengo T. Joshi V. Kumar P. S. (2013). Deep sequencing identifies ethnicity-specific bacterial signatures in the oral microbiome. PLoS ONE 8:e77287. 10.1371/journal.pone.0077287 24194878 PMC3806732

[B61] Moossavi S. (2014). The necessity for an Iranian gut microbiome initiative. Middle East J. Dig. Dis. 6, 109–110. 24872871 PMC4034665

[B62] Morris A. Beck J. M. Schloss P. D. Campbell T. B. Crothers K. Curtis J. L.. (2013). Comparison of the respiratory microbiome in healthy nonsmokers and smokers. Am. J. Respir. Crit. Care Med. 187, 1067–1075. 10.1164/rccm.201210-1913OC 23491408 PMC3734620

[B63] Morton E. R. Lynch J. Froment A. Lafosse S. Heyer E. Przeworski M.. (2015). Variation in rural african gut microbiota is strongly correlated with colonization by entamoeba and subsistence. PLoS Genet. 11:e1005658. 10.1371/journal.pgen.1005658 26619199 PMC4664238

[B64] Nam Y. D. Jung M. J. Roh S. W. Kim M. S. Bae J. W. (2011). Comparative analysis of Korean human gut microbiota by barcoded pyrosequencing. PLoS ONE 6:e22109. 10.1371/journal.pone.0022109 21829445 PMC3146482

[B65] Nasidze I. Li J. Quinque D. Tang K. Stoneking M. (2009). Global diversity in the human salivary microbiome. Genome Res. 19, 636–643. 10.1101/gr.084616.108 19251737 PMC2665782

[B66] Nasidze I. Li J. Schroeder R. Creasey J. L. Li M. Stoneking M. (2011). High diversity of the saliva microbiome in Batwa Pygmies. PLoS ONE 6:e23352. 10.1371/journal.pone.0023352 21858083 PMC3156759

[B67] Nishijima S. Suda W. Oshima K. Kim S. W. Hirose Y. Morita H.. (2016). The gut microbiome of healthy Japanese and its microbial and functional uniqueness. DNA Res. 23, 125–133. 10.1093/dnares/dsw002 26951067 PMC4833420

[B68] Noble W. C. (1984). Skin microbiology: coming of age. J. Med. Microbiol. 17, 1–12. 10.1099/00222615-17-1-1 6229637

[B69] Nomura I. Gao B. Boguniewicz M. Darst M. A. Travers J. B. Leung D. Y. (2003a). Distinct patterns of gene expression in the skin lesions of atopic dermatitis and psoriasis: a gene microarray analysis. J. Allergy Clin. Immunol. 112, 1195–1202. 10.1016/j.jaci.2003.08.049 14657882

[B70] Nomura I. Goleva E. Howell M. D. Hamid Q. A. Ong P. Y. Hall C. F.. (2003b). Cytokine milieu of atopic dermatitis, as compared to psoriasis, skin prevents induction of innate immune response genes. J. Immunol. 171, 3262–3269. 10.4049/jimmunol.171.6.3262 12960356

[B71] Obregon-Tito A. J. Tito R. Y. Metcalf J. Sankaranarayanan K. Clemente J. C. Ursell L. K.. (2015). Subsistence strategies in traditional societies distinguish gut microbiomes. Nat. Commun. 6:6505. 10.1038/ncomms7505 25807110 PMC4386023

[B72] O'Keefe S. J. Chung D. Mahmoud N. Sepulveda A. R. Manafe M. Arch J.. (2007). Why do African Americans get more colon cancer than Native Africans? J. Nutr. 137, 175S–182S. 17182822 10.1093/jn/137.1.175S

[B73] O'Keefe S. J. Li J. V. Lahti L. Ou J. Carbonero F. Mohammed K.. (2015). Fat, fibre and cancer risk in African Americans and rural Africans. Nat. Commun. 6:6342. 10.1038/ncomms7342 25919227 PMC4415091

[B74] Ong P. Y. Ohtake T. Brandt C. Strickland I. Boguniewicz M. Ganz T.. (2002). Endogenous antimicrobial peptides and skin infections in atopic dermatitis. N. Engl. J. Med. 347, 1151–1160. 10.1056/NEJMoa021481 12374875

[B75] Ou J. Carbonero F. Zoetendal E. G. DeLany J. P. Wang M. Newton K.. (2013). Diet, microbiota, and microbial metabolites in colon cancer risk in rural Africans and African Americans. Am. J. Clin. Nutr. 98, 111–120. 10.3945/ajcn.112.056689 23719549 PMC3683814

[B76] Peek R. M. Jr. Blaser M. J. (2002). Helicobacter pylori and gastrointestinal tract adenocarcinomas. Nat. Rev. Cancer 2, 28–37. 10.1038/nrc703 11902583

[B77] Qin J. Li R. Raes J. Arumugam M. Burgdorf K. S. Manichanh C.. (2010). A human gut microbial gene catalogue established by metagenomic sequencing. Nature 464, 59–65. 10.1038/nature08821 20203603 PMC3779803

[B78] Qin J. Li Y. Cai Z. Li S. Zhu J. Zhang F.. (2012). A metagenome-wide association study of gut microbiota in type 2 diabetes. Nature 490, 55–60. 10.1038/nature11450 23023125

[B79] Rampelli S. Schnorr S. L. Consolandi C. Turroni S. Severgnini M. Peano P.. (2015). Metagenome sequencing of the hadza hunter-gatherer gut microbiota. Curr. Biol. 25, 1682–1693. 10.1016/j.cub.2015.04.055 25981789

[B80] Ravel J. Gajer P. Abdo Z. Schneider G. M. Koenig S. S. McCulle S. L.. (2011). Vaginal microbiome of reproductive-age women. Proc. Natl. Acad. Sci. U.S.A. 108(Suppl. 1), 4680–4687. 10.1073/pnas.1002611107 20534435 PMC3063603

[B81] Rehman A. Rausch P. Wang J. Skieceviciene J. Kiudelis G. Bhagalia K.. (2016). Geographical patterns of the standing and active human gut microbiome in health and IBD. Gut 65, 238–248. 10.1136/gutjnl-2014-308341 25567118

[B82] Roth R. R. James W. D. (1989). Microbiology of the skin: resident flora, ecology, infection. J. Am. Acad. Dermatol. 20, 367–390. 10.1016/S0190-9622(89)70048-7 2645319

[B83] Sankaranarayanan K. Ozga A. T. Warinner C. Tito R. Y. Obregon-Tito A. J. Xu J.. (2015). Gut microbiome diversity among cheyenne and arapaho individuals from Western Oklahoma. Curr. Biol. 25, 3161–3169. 10.1016/j.cub.2015.10.060 26671671 PMC4703035

[B84] Schnorr S. L. Candela M. Rampelli S. Centanni M. Consolandi C. Basaglia G.. (2014). Gut microbiome of the Hadza hunter-gatherers. Nat. Commun. 5:3654. 10.1038/ncomms4654 24736369 PMC3996546

[B85] Stearns J. C. Davidson C. J. McKeon S. Whelan F. J. Fontes M. E. Schryvers A. B.. (2015). Culture and molecular-based profiles show shifts in bacterial communities of the upper respiratory tract that occur with age. ISME J. 9, 1246–1259. 10.1038/ismej.2014.250 25575312 PMC4409167

[B86] Tagami H. (2008). Location-related differences in structure and function of the stratum corneum with special emphasis on those of the facial skin. Int. J. Cosmet. Sci. 30, 413–434. 10.1111/j.1468-2494.2008.00459.x 19099543

[B87] Tana C. Umesaki Y. Imaoka A. Handa T. Kanazawa M. Fukudo S. (2010). Altered profiles of intestinal microbiota and organic acids may be the origin of symptoms in irritable bowel syndrome. Neurogastroenterol. Motil. 22, e114–e515. 10.1111/j.1365-2982.2009.01427.x 19903265

[B88] Tarabichi Y. Li K. Hu S. Nguyen C. Wang X. Elashoff D.. (2015). The administration of intranasal live attenuated influenza vaccine induces changes in the nasal microbiota and nasal epithelium gene expression profiles. Microbiome 3:74. 10.1186/s40168-015-0133-2 26667497 PMC4678663

[B89] Turnbaugh P. J. Ley R. E. Hamady M. Fraser-Liggett C. M. Knight R. Gordon J. I. (2007). The human microbiome project. Nature 449, 804–810. 10.1038/nature06244 17943116 PMC3709439

[B90] Turnbaugh P. J. Ley R. E. Mahowald M. A. Magrini V. Mardis E. R. Gordon J. I. (2006). An obesity-associated gut microbiome with increased capacity for energy harvest. Nature 444, 1027–1031. 10.1038/nature05414 17183312

[B91] Tyakht A. V. Kostryukova E. S. Popenko A. S. Belenikin M. S. Pavlenko A. V. Larin A. K.. (2013). Human gut microbiota community structures in urban and rural populations in Russia. Nat. Commun. 4:2469. 10.1038/ncomms3469 24036685 PMC3778515

[B92] Van Treuren W. Ponnusamy L. Brinkerhoff R. J. Gonzalez A. Parobek C. M. Juliano J. J.. (2015). Variation in the microbiota of ixodes ticks with regard to geography, species, and sex. Appl. Environ. Microbiol. 81, 6200–6209. 10.1128/AEM.01562-15 26150449 PMC4542252

[B93] Vebo H. C. Karlsson M. K. Avershina E. Finnby L. Rudi K. (2016). Bead-beating artefacts in the Bacteroidetes to Firmicutes ratio of the human stool metagenome. J. Microbiol. Methods 129, 78–80. 10.1016/j.mimet.2016.08.005 27498349

[B94] Verstraelen H. Vilchez-Vargas R. Desimpel F. Jauregui R. Vankeirsbilck N. Weyers S.. (2016). Characterisation of the human uterine microbiome in non-pregnant women through deep sequencing of the V1-2 region of the 16S rRNA gene. PeerJ 4:e1602. 10.7717/peerj.1602 26823997 PMC4730988

[B95] Wade W. G. (2013). The oral microbiome in health and disease. Pharmacol. Res. 69, 137–143. 10.1016/j.phrs.2012.11.006 23201354

[B96] Walker A. W. Martin J. C. Scott P. Parkhill J. Flint H. J. Scott K. P. (2015). 16S rRNA gene-based profiling of the human infant gut microbiota is strongly influenced by sample processing and PCR primer choice. Microbiome 3:26. 10.1186/s40168-015-0087-4 26120470 PMC4482049

[B97] Wang Z. Klipfell E. Bennett B. J. Koeth R. Levison B. S. Dugar B.. (2011). Gut flora metabolism of phosphatidylcholine promotes cardiovascular disease. Nature 472, 57–63. 10.1038/nature09922 21475195 PMC3086762

[B98] Yap G. C. Chee K. K. Hong P. Y. Lay C. Satria C. D. Sumadiono S.. (2011). Evaluation of stool microbiota signatures in two cohorts of Asian (Singapore and Indonesia) newborns at risk of atopy. BMC Microbiol. 11:193. 10.1186/1471-2180-11-193 21875444 PMC3171725

[B99] Yatsunenko T. Rey F. E. Manary M. J. Trehan I. Dominguez-Bello M. G. Contreras M.. (2012). Human gut microbiome viewed across age and geography. Nature 486, 222–227. 10.1038/nature11053 22699611 PMC3376388

[B100] Yi H. Yong D. Lee K. Cho Y. J. Chun J. (2014). Profiling bacterial community in upper respiratory tracts. BMC Infect. Dis. 14:583. 10.1186/s12879-014-0583-3 25391813 PMC4236460

[B101] Ying S. Zeng D. N. Chi L. Tan Y. Galzote C. Cardona C.. (2015). The influence of age and gender on skin-associated microbial communities in urban and rural human populations. PLoS ONE 10:e0141842. 10.1371/journal.pone.0141842 26510185 PMC4624872

[B102] Zeller G. Tap J. Voigt A. Y. Sunagawa S. Kultima J. R. Costea P. I.. (2014). Potential of fecal microbiota for early-stage detection of colorectal cancer. Mol. Syst. Biol. 10:766. 10.15252/msb.20145645 25432777 PMC4299606

[B103] Zhernakova A. Kurilshikov A. Bonder M. J. Tigchelaar E. F. Schirmer M. Vatanen T.. (2016). Population-based metagenomics analysis reveals markers for gut microbiome composition and diversity. Science 352, 565–569. 10.1126/science.aad3369 27126040 PMC5240844

[B104] Zhou X. Brown C. J. Abdo Z. Davis C. C. Hansmann M. A. Joyce P.. (2007). Differences in the composition of vaginal microbial communities found in healthy Caucasian and black women. ISME J. 1:121–133. 10.1038/ismej.2007.12 18043622

[B105] Zhou X. Hansmann M. A. Davis C. C. Suzuki H. Brown C. J. Schutte U.. (2010). The vaginal bacterial communities of Japanese women resemble those of women in other racial groups. FEMS Immunol. Med. Microbiol. 58, 169–181. 10.1111/j.1574-695X.2009.00618.x 19912342 PMC2868947

