# Supplementary material for: Geography, Ethnicity or Subsistence-Specific Variations in Human Microbiome Composition and Diversity
Source: Front Microbiol. 2017 Jun 23;8:1162. doi: 10.3389/fmicb.2017.01162 (PMC5481955; doi:10.3389/fmicb.2017.01162)
Supplement: Supplementary file 1 [file Table1.PDF]

**Table S1: Relative abundance (%) of core microbiome in gut derived from 12 different populations around the world**

| | Malawi[99] | Venezuela[99] | Peru[71] | USA[99&71] | China[78] | Denmark[77] | Spain[77] | Russia[91] | Sweden[49] | France[102] | Austria[32] | Japan[67] |
|---|---|---|---|---|---|---|---|---|---|---|---|---|
| *Bacteroides* | 3.28 | 3.96 | 1.62 | 38.38 | 39.70 | 20.51 | 23.33 | 8.47 | 8.16 | 13.43 | 6.43 | 11.37 |
| *Prevotella* | 32.65 | 24.89 | 14.85 | 3.94 | 9.15 | 7.61 | 7.41 | 10.64 | 0.86 | 2.64 | 0.38 | 2.38 |
| *Eubacterium* | 8.31 | 4.42 | 5.55 | 4.71 | 9.42 | 7.53 | 9.51 | 12.52 | 9.90 | 9.77 | 10.05 | 6.55 |
| *Clostridium* | 8.51 | 8.57 | 11.19 | 3.96 | 5.77 | 12.05 | 8.86 | 10.76 | 10.31 | 7.55 | 5.63 | 2.28 |
| *Faecalibacterium* | 4.97 | 6.93 | 10.19 | 4.94 | 5.07 | 4.76 | 4.21 | 4.05 | 7.88 | 7.20 | 5.44 | 5.74 |
| *unclassified Firmicutes* | 6.05 | 7.08 | 8.39 | 4.07 | 3.21 | 9.12 | 5.67 | 5.56 | 8.66 | 6.12 | 5.81 | 1.51 |
| *Ruminococcus* | 4.89 | 3.47 | 8.18 | 5.48 | 2.02 | 3.32 | 5.80 | 6.48 | 8.16 | 7.82 | 9.16 | 5.57 |
| *Blautia* | 3.84 | 2.05 | 2.99 | 3.13 | 2.18 | 1.96 | 4.91 | 5.63 | 6.99 | 5.81 | 12.87 | 16.69 |
| *Alistipes* | 3.82 | 7.23 | 9.57 | 6.25 | 3.62 | 5.59 | 3.61 | 1.97 | 4.29 | 3.59 | 2.67 | 0.67 |
| *Bifidobacterium* | 0.06 | 1.90 | 0.71 | 1.61 | 0.54 | 0.70 | 1.76 | 1.12 | 2.30 | 7.24 | 10.86 | 17.93 |
| *Roseburia* | 3.37 | 1.81 | 2.22 | 2.35 | 3.71 | 4.99 | 3.98 | 7.14 | 4.86 | 3.84 | 2.94 | 2.15 |
| *Coprococcus* | 0.89 | 0.60 | 1.04 | 0.55 | 0.59 | 1.62 | 1.56 | 4.16 | 2.36 | 1.96 | 1.66 | 1.39 |
| *Escherichia* | 0.22 | 4.32 | 1.22 | 0.88 | 1.02 | 0.70 | 0.11 | 1.35 | 2.71 | 1.19 | 1.15 | 0.52 |
| *Parabacteroides* | 0.17 | 0.11 | 0.09 | 3.47 | 2.04 | 1.97 | 1.94 | 0.83 | 0.79 | 1.30 | 0.49 | 2.02 |
| *Dorea* | 0.86 | 0.77 | 0.54 | 0.58 | 0.41 | 0.59 | 1.27 | 2.02 | 1.91 | 1.42 | 2.43 | 2.07 |
| *Dialister* | 2.61 | 0.91 | 1.79 | 1.15 | 0.36 | 1.34 | 0.89 | 1.01 | 1.36 | 0.72 | 1.11 | 0.21 |
| *Anaerostipes* | 0.22 | 0.29 | 0.14 | 0.43 | 0.41 | 0.45 | 1.13 | 0.84 | 1.42 | 1.33 | 4.45 | 1.96 |
| *Streptococcus* | 1.45 | 1.89 | 0.29 | 0.81 | 0.49 | 0.13 | 0.53 | 1.21 | 0.92 | 1.39 | 1.48 | 2.22 |
| *Butyrivibrio* | 0.97 | 0.87 | 1.06 | 0.28 | 0.17 | 2.22 | 1.11 | 1.59 | 1.67 | 1.18 | 0.31 | 0.03 |
| *Collinsella* | 0.23 | 0.45 | 1.00 | 0.38 | 0.06 | 0.18 | 0.45 | 0.14 | 0.93 | 1.32 | 2.46 | 3.01 |
| *Phascolarctobacterium* | 0.43 | 2.09 | 0.98 | 0.59 | 1.23 | 0.85 | 0.95 | 0.43 | 0.45 | 0.68 | 0.31 | 0.77 |
| *Clostridiales* | 0.33 | 0.29 | 0.45 | 0.73 | 0.68 | 0.74 | 0.79 | 0.68 | 0.68 | 0.88 | 0.97 | 1.15 |
| *Methanobrevibacter* | 0.47 | 0.35 | 1.49 | 0.10 | 0.01 | 0.12 | 1.07 | 1.54 | 1.32 | 0.51 | 0.61 | 0.00 |
| *Akkermansia* | 0.00 | 0.01 | 0.29 | 0.74 | 0.21 | 0.76 | 0.48 | 1.05 | 1.23 | 0.97 | 1.54 | 0.15 |
| *Ruminiclostridium* | 0.10 | 0.16 | 0.18 | 0.81 | 0.24 | 1.20 | 0.50 | 0.41 | 1.21 | 0.62 | 0.47 | 0.43 |

***Superscripted numbers:** *references from the main text of manuscript*
