# Supplementary material for: Geography, Ethnicity or Subsistence-Specific Variations in Human Microbiome Composition and Diversity
Source: Front Microbiol. 2017 Jun 23;8:1162. doi: 10.3389/fmicb.2017.01162 (PMC5481955; doi:10.3389/fmicb.2017.01162)
Supplement: Supplementary file 2 [file Table2.PDF]

Table S2: Relative abundance (%) of core microbiome in saliva derived from 15 different populations around the world

	Argentina[65]	Bolivia[65]	USA-California[65]	USA-Louisiana[65]	USA-Alaska[56]	Germany[65]	Poland[65]	Georgia[65]	Turkey[65]	Congo[65]	South Africa[65]	China[65]	Philippines[65]	Uganda-Batwa Pygmy[66]	Sierra Leone-Farming[66]
Actinomyces	1.77	0.25	0.93	1.52	1.6	1.99	2.49	0.93	1.31	0.93	2.33	0.7	2.88	0.69	0.78
Aggregatibacter	0.42	0.33	0.42	0.63	0.45	0.87	0.51	0.51	0.17	0.51	0.24	0.96	0.51	1.15	0.17
Campylobacter	0.67	0.49	0.42	0.36	0.4	1.39	1.2	0.51	0.96	0.08	0.64	0.52	0.34	0.72	0.06
Fusobacterium	2.19	1.56	1.02	1.7	2.65	2.51	1.8	2.11	5.33	1.36	1.76	2.79	1.78	2.89	2.01
Gemella	0.51	1.31	1.35	2.15	2.25	0.26	0.43	0.34	0.52	0.85	0.72	0.78	0.42	0.55	0.03
Granulicatella	1.26	0.74	1.61	1.7	1.65	1.47	0.51	2.11	1.22	1.78	0.72	1.83	1.61	1.70	0.57
Haemophilus	7.74	4.91	6.85	9.42	4.825	7.45	4.97	7.08	4.63	1.95	4.57	6.35	4.23	3.38	2.03
Leptotrichia	2.27	1.47	0.51	1.79	0.65	1.65	1.8	2.02	2.1	0.25	1.04	1.74	1.44	2.85	0.70
Neisseria	12.46	5.16	6.18	11.75	2.325	6.58	4.37	9.27	4.98	2.89	4.33	19.67	7.53	5.39	3.29
Oribacterium	1.09	0.66	1.02	0.36	0.575	0.52	0.94	0.51	0.96	1.36	1.2	0.52	0.25	0.44	0.06
Porphyromonas	2.1	0.98	1.27	0.81	1.1	3.38	0.77	3.46	3.76	2.38	2.57	4.26	2.45	4.57	1.49
Prevotella	20.71	14.5	13.79	5.65	28.325	16.36	26.65	24.11	28.47	5.18	19.81	13.14	16.92	13.65	6.50
Rothia	5.98	1.88	7.28	7.26	10.5	14.63	2.91	2.45	3.93	2.72	3.21	2	3.98	3.30	1.78
Streptococcus	15.91	23.34	28	39.19	26	26.32	19.37	14.42	18.78	16.89	23.98	25.33	21.24	13.01	12.33
Veillonella	13.55	8.27	12.1	5.11	8.325	5.89	12.43	7.08	9.78	2.21	12.75	2.52	11.17	3.71	1.30

***Superscripted numbers:** *references from the main text of manuscript*
