# Supplementary material for: Geography, Ethnicity or Subsistence-Specific Variations in Human Microbiome Composition and Diversity
Source: Front Microbiol. 2017 Jun 23;8:1162. doi: 10.3389/fmicb.2017.01162 (PMC5481955; doi:10.3389/fmicb.2017.01162)
Supplement: Supplementary file 3 [file Table3.PDF]

Table S3: Relative abundance (%) of core vaginal microbiome derived from 7 different populations

	**Belgium**[94]	**USA-Asian**[80]	**USA-Black**[80]	**USA-Hispanic**[80]	**USA-White**[80]	**Canada**[2]	**Japan**[105]
Lactobacillus	16.67	76.95	60.32	62.06	83.93	69.89	79.16
Prevotella	3.82	3.86	8.85	8.13	3.42	3.90	1.73
Atopobium	0.84	1.25	2.73	3.80	0.89	1.08	6.03
Megasphaera	0.28	1.07	5.84	3.79	1.70	2.01	0.56
Dialister	0.18	0.80	1.90	2.04	0.68	0.27	0.51
Others	78.22	16.07	20.36	20.18	9.38	22.85	12.01

*__Superscripted numbers:__ *references from the main text of manuscript*
