# Supplementary material for: Geography, Ethnicity or Subsistence-Specific Variations in Human Microbiome Composition and Diversity
Source: Front Microbiol. 2017 Jun 23;8:1162. doi: 10.3389/fmicb.2017.01162 (PMC5481955; doi:10.3389/fmicb.2017.01162)
Supplement: Supplementary file 4 [file Table4.PDF]

Table S4 – Methodological information (DNA extraction, Sequencer, Primers) for studies included in this review

Citation[#]	Samples	HVR*	FP**	RP***	Sequencer	DNA extraction method	Instrument for DNA extraction
12	Fecal	V3-V4	515	E.coli 907-924	454 GS FLX	ML	QIAamp DNA Stool Mini Kit
23	Fecal	V5-V6	784	1061	454 GS FLX	EL	RNAlater (Qiagen)
30	Fecal	FISH	-	-	-	EL	-
38	Fecal	V1-V3	27	534	454 GS FLX	ML	MoBio Ultraclean Soil Kit
**49**	Fecal	**-**	**-**	**-**	**HiSeq2000**	**-**	**NA**
50	Fecal	V4	515	806	454 GS FLX	ML/CL/EL	FastDNA® Kit
57	Fecal	V3–V5	PRK341	PRK806	SOLiDv4	ML	QIAamp DNA stool Mini Kit
63	Fecal	V5–V6	V5F	V6R	MiSeq	ML	PowerFecal DNA Isolation Kit
64	Fecal	V1-V2	9	541	454 GS FLX	ML	DNA<br>Isolation Kit
**67**	Fecal	**-**	**-**	**-**	**454 GS FLX, Ion Torrent PGM, & MiSeq**	**EL**	**enzymatic lysis method using lysozyme**
**71**	Fecal	-	-	**-**	**Illumina**	**ML**	**PowerSoil DNA Isolation Kit**
75	Fecal	-	519	926	454 GS FLX	ML	QIAamp DNA Stool Mini Kit (bead-beating by using the FastPrep-24 System)
81	Fecal	V1–V2	27	338	454 GS FLX		Qiagen Allprep DNA/RNA
83	Fecal	V4	-	-	MiSeq	ML	PowerMicrobiome RNA Isolation Kit (bead-beating)
84	Fecal	V4	520	802	454 GS FLX	ML	QIAamp DNA Stool Mini Kit (QIAGEN) (bead-beating by using the FastPrep-24 System)
**91**	Fecal	-	-	**-**	**SOLiDv4**	**EL**	**BeadBeater 16**
99	Fecal	V4			Illumina		
32	Fecal	-	-	-	Illumina	NA	-
77	Fecal	-	63	1387	Illumina	EL	Qiagen plasmid mini kit
78	Fecal	-	63	1387	Illumina	EL	Qiagen plasmid mini kit
102	Fecal	V3-V4	515	806	Illumina	ML	GNOME DNA Isolation Kit
3	Respiratory tract	V1-V2	27	534	454 GS FLX	EL	bead beating and phenol-chloroform extraction methods

7	Respiratory tract	V3-V5	8	357	454 GS FLX	ML	PowerSoil DNA kit(Mini BeadBeater-8)
13	Respiratory tract	V1-V2	27	338	454 GS FLX	ML	PowerSoil DNA Isolation Kit
16	Respiratory tract	V1-V2	8	357	454 GS FLX	ML	PowerSoil DNA isolation kit (bead-beating)
25	Respiratory tract	V5–V7	785	1061	454 GS FLX	EL	extraction kit protocol (bead-beating)
27	Respiratory tract	V4	515	806	MiSeq	ML	UltraClean fecal DNA bead tubes
28	Respiratory tract	V4	515	806	MiSeq	ML	UltraClean fecal DNA bead tubes
62	Respiratory tract	V1–V3 V3-V5	27/357	534/926	454 GS FLX	ML	PowerSoil DNA Isolation Kit (bead-beating)
85	Respiratory tract	V3	8	926	MiSeq	ML/EL	mechanical and enzymatic lysis (bead-beating)
88	Respiratory tract	V1–V3 V3-V5	-	-	454 GS FLX	ML	PowerSoil DNA Isolation Kit
26	Saliva	-	F24/Y36	F24/Y36	ABI Prism cycle sequencing kit	ML	UltraClean microbial DNA isolation kit
56	Saliva	V1- V2	27	338	454 GS FLX	EL	Salting out
60	Saliva	V1–V3 V7–V9	A17 & 317	A17 & 317	454 GS FLX	ML	Qiagen MiniAmp kit
65 & 66	Saliva	V1-V2	-	-	454 GS FLX	-	-
10	Skin	V2	27	338	454 GS FLX	ML	PowerSoil DNA Isolation kit
45	Skin	V3-V5	343	926	454 GS FLX	ML	PowerMax Soil DNA Isolation Kit
54	Skin	V4	515	806	MiSeq	ML	PowerSoil DNA isolation kit (bead-beating)
101	Skin	V1-V2	27FYM	536RK	454 GS FLX	ML	PowerSoil DNA Isolation Kit (bead-beating)
2	Vaginal	V3	GPO1	MGSO	454 GS FLX	ML	MagMAX Total Nucleic Acid Isolation Kit
33	Vaginal	V1-V3	Fwd-P1	Rev1B	454 GS FLX	ML	Powersoil kit
80	Vaginal	V1-V2	27	338	454 GS FLX	EL/ML	QIAamp DNA Mini Kit (bead-beating)
94	Vaginal	V1-V2	27	338	MiSeq	ML/CL/EL	FastPrep R □24
104	Vaginal	V1-V3	Fwd-P1 & P2	Rev1B	454 GS FLX	ML	Powersoil kit
105	Vaginal	V1-V3	Fwd-P1 & P2	Rev1B	454 GS FLX	ML	Powersoil kit

****HVR**: Hyper Variable Region of 16S rRNA; ****FP**: Forward Primer; *****RP**: Reverse Primer; All studies are based on 16S rRNA Gene Amplification except bold (whole shotgun); # references from the main text of manuscript*
