# Supplementary material for: Geography, Ethnicity or Subsistence-Specific Variations in Human Microbiome Composition and Diversity
Source: Front Microbiol. 2017 Jun 23;8:1162. doi: 10.3389/fmicb.2017.01162 (PMC5481955; doi:10.3389/fmicb.2017.01162)
Supplement: Supplementary file 5 [file Image1.PDF]

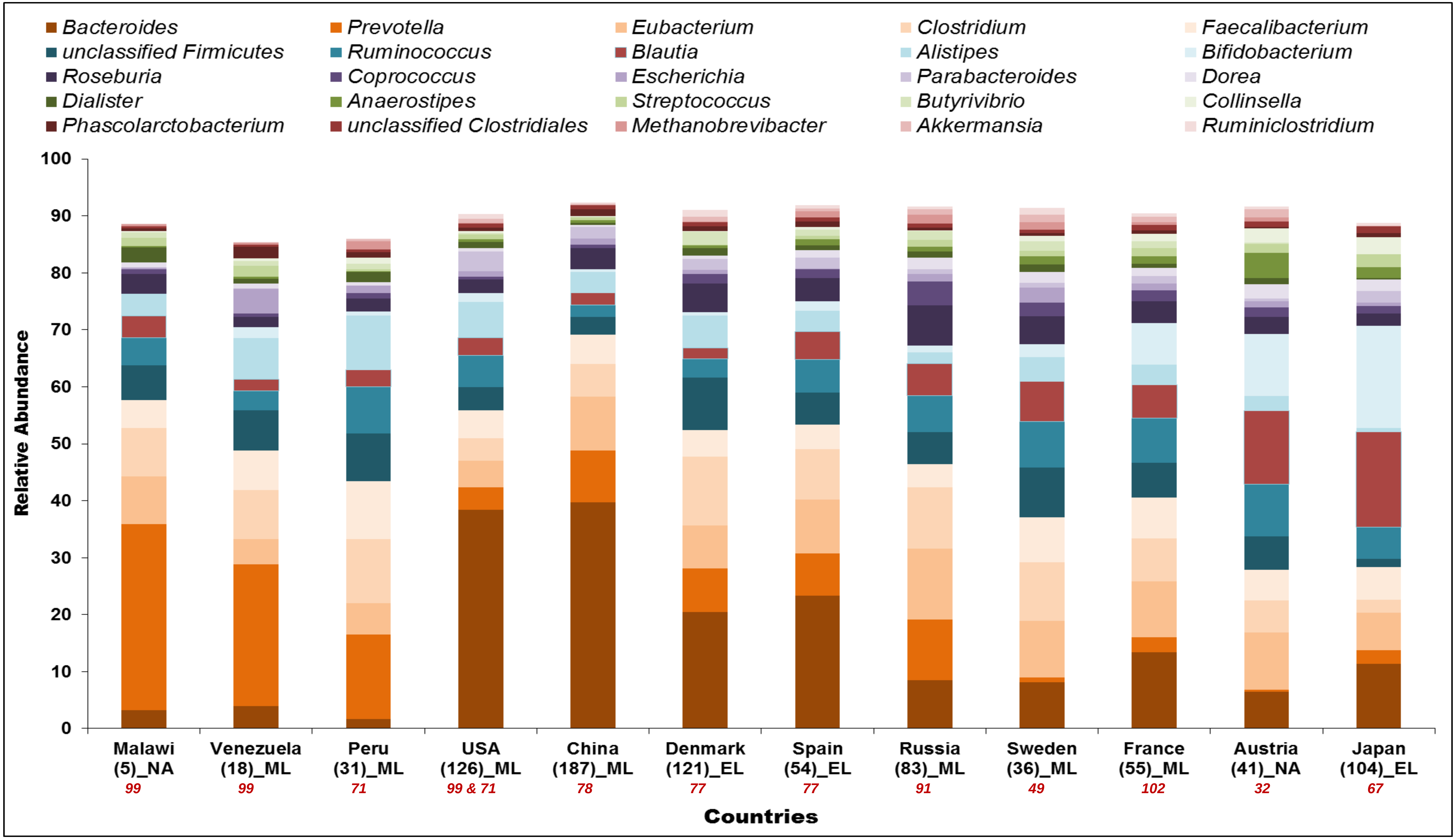

Figure S1: Composition of core gut (stool) microbiota in healthy individuals from 12 different geographical countries/populations
DNA extraction methods: *ML-Mechanical Lysis; EL-Enzymatic Lysis; NA-Information not available;* ***Red color digits:*** *Reference number from the main text of manuscript;* ***Number in parenthesis:*** *Number of individuals used in respective study;* ***Data provided in Table S1; all are fecal samples***
