# Supplementary material for: Geography, Ethnicity or Subsistence-Specific Variations in Human Microbiome Composition and Diversity
Source: Front Microbiol. 2017 Jun 23;8:1162. doi: 10.3389/fmicb.2017.01162 (PMC5481955; doi:10.3389/fmicb.2017.01162)
Supplement: Supplementary file 6 [file Image2.PDF]

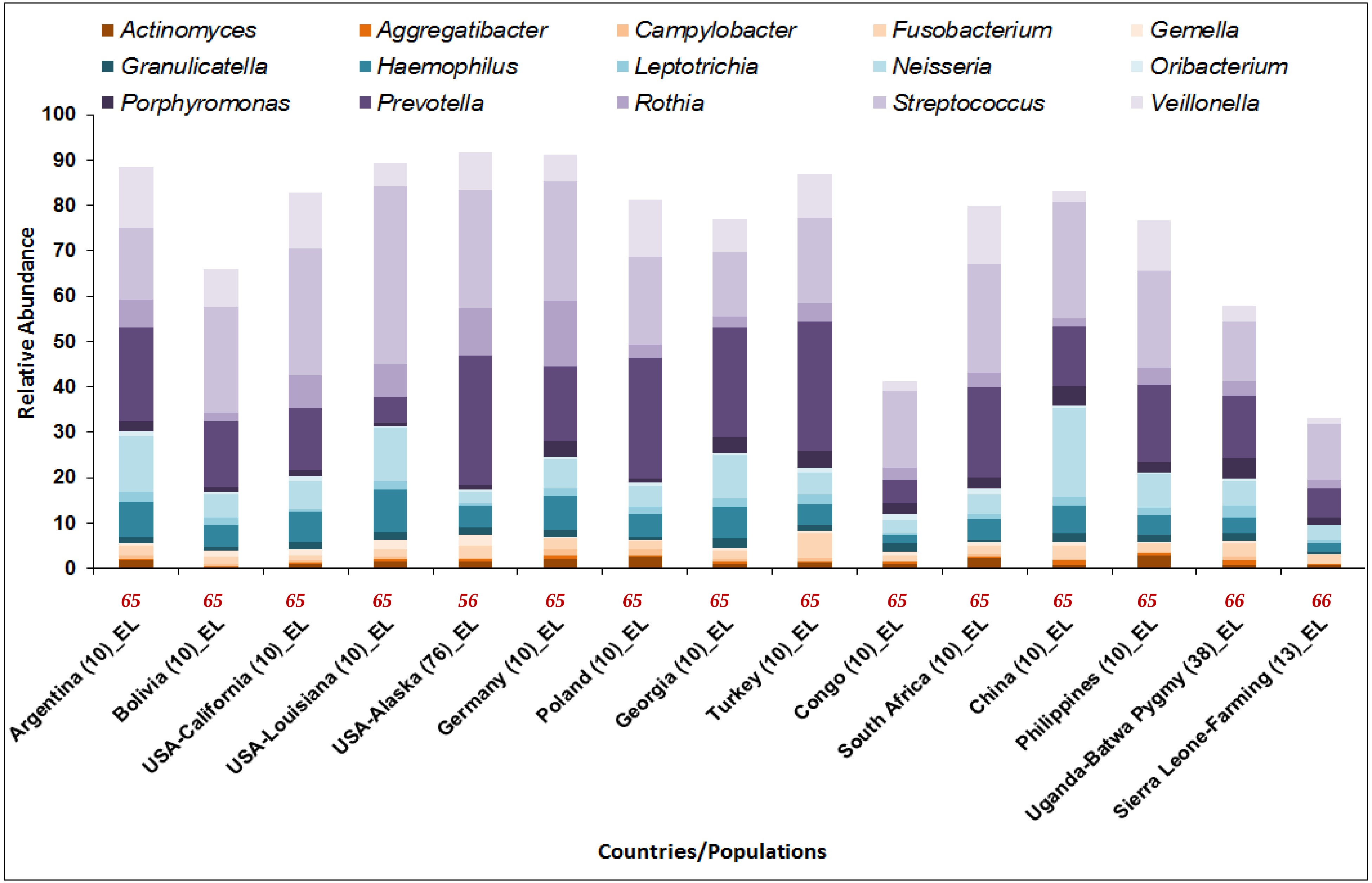

Figure S2: Composition of core microbiota of saliva/oral cavity in healthy individuals from 15 different populations

DNA extraction methods: *EL-Enzymatic Lysis;* ***Red color digits:*** *Reference number from the main text of manuscript;* ***Number in parenthesis:*** *Number of individuals used in respective study;*
Data provided in Table S2; all samples derived from saliva
