# Supplementary material for: Geography, Ethnicity or Subsistence-Specific Variations in Human Microbiome Composition and Diversity
Source: Front Microbiol. 2017 Jun 23;8:1162. doi: 10.3389/fmicb.2017.01162 (PMC5481955; doi:10.3389/fmicb.2017.01162)
Supplement: Supplementary file 7 [file Image3.PDF]

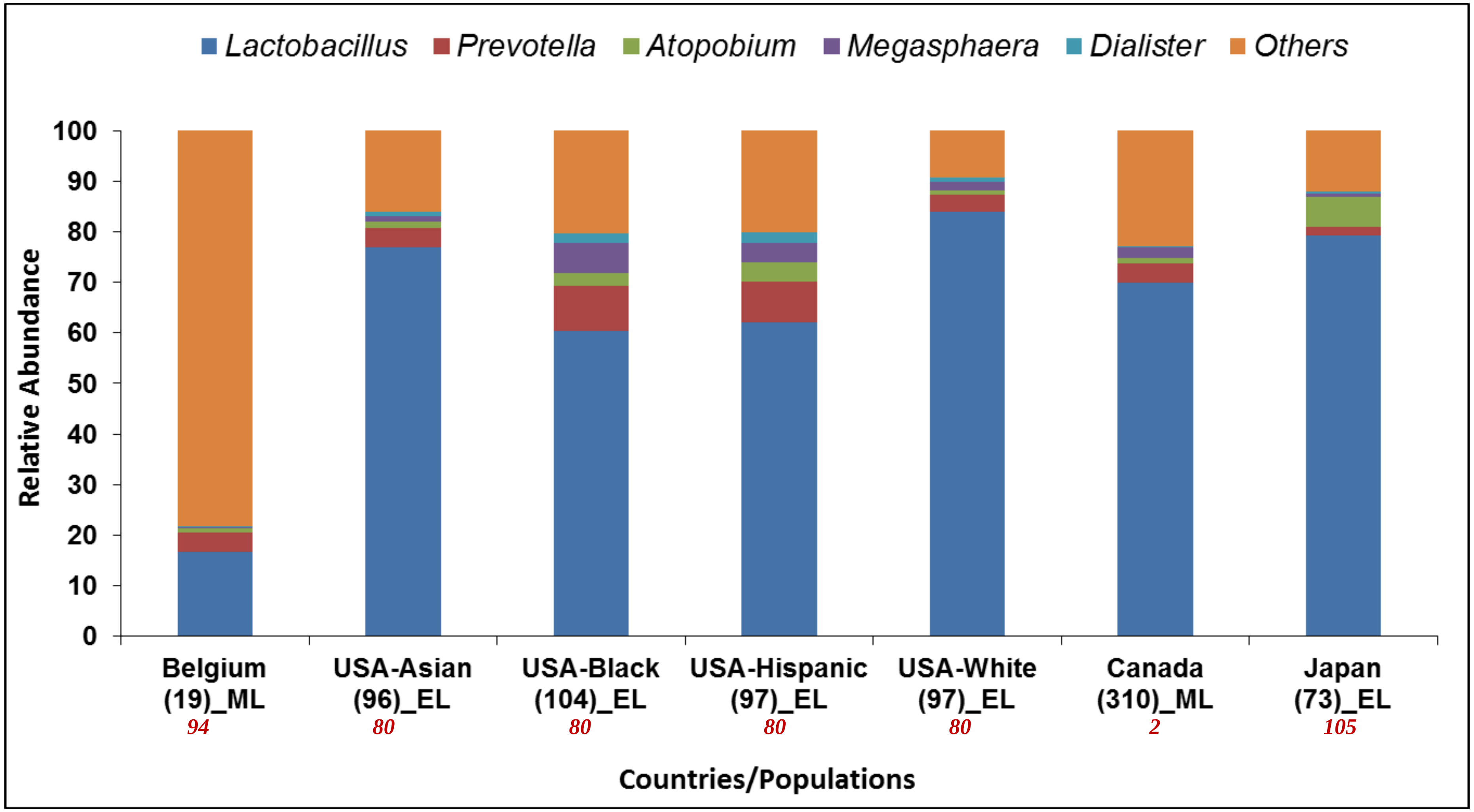

Figure S3: Composition of core vaginal microbiota in healthy individuals from 7 different populations
DNA extraction methods: *ML-Mechanical Lysis; EL-Enzymatic Lysis;* ***Red color digits:*** *Reference number from the main text of manuscript;* ***Number in parenthesis:*** *Number of individuals used in respective study;* ***Data provided in Table S3; all samples derived from vaginal swab***
